# Distinct Roles for Two Chromosome 1 Loci in Ethanol Withdrawal, Consumption, and Conditioned Place Preference

**DOI:** 10.3389/fgene.2018.00323

**Published:** 2018-08-27

**Authors:** Laura B. Kozell, Deaunne L. Denmark, Nicole A. R. Walter, Kari J. Buck

**Affiliations:** Department of Behavioral Neuroscience, Portland Veterans Affairs Medical Center and School of Medicine, Oregon Health and Science University, Portland, OR, United States

**Keywords:** quantitative trait locus (QTL), anxiety, convulsions, consumption, GIRK

## Abstract

We previously identified a region on chromosome 1 that harbor quantitative trait loci (QTLs) with large effects on alcohol withdrawal risk using both chronic and acute models in mice. Here, using newly created and existing QTL interval-specific congenic (ISC) models, we report the first evidence that this region harbors two distinct alcohol withdrawal QTLs (*Alcw1*_1_and *Alcw1*_2_), which underlie 13% and 3–6%, respectively, of the genetic variance in alcohol withdrawal severity measured using the handling-induced convulsion. Our results also precisely localize *Alcw1*_1_ and *Alcw1*_2_ to discreet chromosome regions (syntenic with human 1q23.1–23.3) that encompass a limited number of genes with validated genotype-dependent transcript expression and/or non-synonymous sequence variation that may underlie QTL phenotypic effects. ISC analyses also implicate *Alcw1*_1_and *Alcw1*_2_ in withdrawal-induced anxiety-like behavior, representing the first evidence for their broader roles in alcohol withdrawal beyond convulsions; but detect no evidence for *Alcw1*_2_ involvement in ethanol conditioned place preference (CPP) or consumption. Our data point to high-quality candidates for *Alcw1*_2_, including genes involved in mitochondrial respiration, spatial buffering, and neural plasticity, and to *Kcnj9* as a high-quality candidate for *Alcw1*_1_. Our studies are the first to show, using two null mutant models on different genetic backgrounds, that *Kcnj9* ^−/−^ mice demonstrate significantly less severe alcohol withdrawal than wildtype littermates using acute and repeated exposure paradigms. We also demonstrate that *Kcnj9* ^−/−^ voluntarily consume significantly more alcohol (20%, two-bottle choice) than wildtype littermates. Taken together with evidence implicating *Kcnj9* in ethanol CPP, our results support a broad role for this locus in ethanol reward and withdrawal phenotypes. In summary, our results demonstrate two distinct chromosome 1 QTLs that significantly affect risk for ethanol withdrawal, and point to their distinct unique roles in alcohol reward phenotypes.

## Introduction

Abuse of alcohol, prescription and other sedative-hypnotic drugs is among the top five health problems identified in the U.S. (Office of National Drug Control Policy, 2004). Alcohol dependence (alcoholism) and abuse affect up to 30% of Americans (Hasin et al., 2007) and complicate most chronic illnesses. Alcohol dependence is also among the most highly heritable addictive disorders (Goldman et al., 2005). However, alcoholism is a heterogeneous disorder with a complex interaction between genetic and environmental factors, making conclusive identification of genetic determinants difficult to elucidate (Ducci and Goldman, 2012). This continues to hamper development of effective therapeutic and prevention strategies.

Although animal models cannot duplicate alcoholism, models for specific factors (e.g., withdrawal and reward phenotypes) have proven useful for identifying potential determinants of liability in humans. Withdrawal is a hallmark of alcohol physiological dependence, and constitutes a motivational force that can maintain the cycle of use and abuse (Little et al., 2005). The handling-induced convulsion (HIC) is a robust measure of CNS hyperexcitability in mice, and a sensitive measure of alcohol withdrawal using acute, repeated, and chronic alcohol exposure models (Goldstein and Pal, 1971; Kosobud and Crabbe, 1986; Crabbe et al., 1991; Metten et al., 2007; Chen et al., 2008). Alcohol withdrawal convulsions occur in all species tested, including humans (Friedman, 1980), and have a clear genetic contribution (Goldstein, 1973; Metten and Crabbe, 1999; Lutz et al., 2006). We previously mapped significant quantitative trait loci (QTLs) with large effects on predisposition to physiological dependence and associated withdrawal convulsions following chronic and acute alcohol exposure in mice (Buck et al., 1997, 2002) to a broad region of chromosome 1.

High resolution QTL mapping is crucial to progress toward identification of the genes that underlie QTL phenotypic effects and, just as importantly, to assess potential pleiotropic effects. One of the most powerful strategies to precisely map a QTL employs interval-specific congenic (ISC) models (Darvasi, 1997; Fehr et al., 2002; Shirley et al., 2004). Because of the near elimination of genetic “noise” from loci elsewhere in the genome, comparisons between congenic and wildtype (WT) animals are invaluable to elucidate QTL actions. Using this strategy, we previously confirmed and mapped a QTL for acute alcohol withdrawal (*Alcw1*) to a maximal 1.7 Mb interval of chromosome 1, and also localized a QTL affecting chronic alcohol withdrawal (*Alcdp1*) to the same 1.7 Mb interval (Kozell et al., 2008). We also mapped a QTL (*Pbw1*) proven to affect both pentobarbital and zolpidem withdrawal to a distinct 0.44 Mb interval of chromosome 1 (Kozell et al., 2009). However, currently, it is unproven whether one, two, or possibly even more distinct QTLs within this QTL rich region in fact affect alcohol withdrawal risk. The present studies report the creation of an ISC model (R3), analyses of which proved invaluable to confirm that at least two distinct alcohol withdrawal QTLs on chromosome 1 (now termed *Alcw1*_1_ and *Alcw1*_2_) exist within the original broad *Alcw1* region (Buck et al., 1997), and we demonstrate that each significantly affects alcohol withdrawal risk.

While some withdrawal signs are genetically correlated with HIC severity (i.e., Kosobud and Crabbe, 1986; Belknap et al., 1987; Feller et al., 1994; tremors, hypoactivity, emotionality), others are not (i.e., tail stiffness; Kosobud and Crabbe, 1986). Thus, assessment of HICs can inform analyses for signs correlated to alcohol withdrawal, but represent only part of a complex syndrome. Furthermore, we and others have noted that the chromosomal region focused on in the present studies is a hotbed for confirmed and putative QTLs for a variety of phenotypes relevant to alcohol actions and many others (Kerns et al., 2005; Denmark and Buck, 2008), including for phenotypes shown to be significantly genetically correlated with risk for alcohol withdrawal convulsions, e.g., ethanol consumption (Metten et al., 2014). Therefore, the present studies also expand upon previous analyses and include additional measures of withdrawal (i.e., anxiety-like behavior) and reward phenotypes (i.e., alcohol self-administration and ethanol conditioned place preference [CPP]) to begin to assess the potential broader actions of *Alcw1*_1_ and *Alcw1*_2_. In summary, our results confirm two alcohol withdrawal QTLs on chromosome 1, and also begin to elucidate their distinct broader roles in alcohol withdrawal and reward behaviors.

## Materials and methods

### Animals

C57BL/6J (B6) and DBA/2J (D2) inbred strain breeders were purchased from the Jackson Laboratory. The four chromosome 1 congenic models were all created in our colony at the Veterinary Medical Unit of the Portland VA Medical Center, and include: a newly created D2.B6 ISC (R3), a recently created D2.B6 ISC (R2; Walter et al., 2017), D2.B6^−*D*1*Mit*206^ (Kozell et al., 2008), and a reciprocal (B6.D2) ISC (R8; Kozell et al., 2008). To maintain our congenic models on an inbred (D2 or B6) genetic background, congenic heterozygotes were backcrossed to background strain animals from the Jackson Laboratory every third generation. *Kcnj9* encodes the G protein-coupled inwardly-rectifying potassium channel subunit 3 (GIRK3). One of the two *Kcnj9* null mutant models (inbred B6 genetic background; Torrecilla et al., 2002) was originally generously provided by Dr. Kevin Wickman, and has been used extensively and maintained in our colony for over 20 generations using a heterozygote (B6-*Kcnj9*^+/−^) x B6-*Kcnj9*^+/−^ breeding strategy, and backcrossing to B6 strain mice every third generation as is required to maintain integrity. The other null mutant *Kcnj9*^−/−^ model [inbred D2 genetic background; Kozell et al., 2009] used in these studies was created and maintained in our colony as above. A total of 1192 mice were behaviorally tested, with males and females used in approximately equal numbers: 670 congenic and appropriate WT animals, and 526 *Kcnj9*^−/−^, *Kcnj9*^+/−^ and WT littermates. Animals were group housed 2–4 per cage by sex. Mouse chow (Purina #5001) and water were available *ad libitum*, and lights were on from 6:00 to 18:00 with the room temperature maintained at 22.0 ± 1.0°C. All procedures were approved by the VA Medical Center and Oregon Health and Science University Institutional Animal Care and Use Committees in accordance with United States Department of Agriculture and United States Public Health Service guidelines.

### Development of D2.B6 ISC strains

We previously showed that a QTL affecting acute withdrawal severity (*Alcw1*) was captured within the introgressed interval of a chromosome 1 congenic strain, D2.B6^−*D*1*Mit*206^ (Kozell et al., 2008). Genotypic analyses delimited its maximal introgressed interval to 151.6-177.5 Mb. Here, we used D2.B6^−*D*1*Mit*206^ as our point of departure to create a novel D2.B6 ISC model (R3). D2.B6^−*D*1*Mit*206^ congenics were crossed to D2 inbred strain mice to yield F_1_ (D2.B6^−*D*1*Mit*206^ X D2) animals, which were then backcrossed to D2 mice. Individual progeny were genotyped using *D1Mit* and single nucleotide polymorphism (SNP) markers within or flanking the acute and chronic alcohol withdrawal QTLs on chromosome 1 (Buck et al., 1997, 2002; http://www.informatics.jax.org/searches/marker_report.cgi) to identify recombinant mice, thereby defining the boundaries of introgressed intervals. Individual recombinants were again backcrossed to D2 strain mice, resulting in multiple offspring with the same recombination. A final intercross used performed to isolate the donor homozygotes, which constitute a finished ISC strain. Congenic and appropriate WT animals are compared in phenotypic analyses to test for QTL “capture” within the differential introgressed congenic interval, as in our previous work (Kozell et al., 2008).

### Alcohol withdrawal HIC phenotypic analyses

Physiological dependence is operationally defined as the manifestation of physical disturbances (withdrawal symptoms) after alcohol administration is suspended. Handling-induced convulsions (HICs), a sensitive index of withdrawal severity (Crabbe et al., 1991; Goldstein and Pal, 1971), were used initially to monitor genetic variation in alcohol withdrawal severity.

#### Acute alcohol model

(McQuarrie and Fingl, 1958) first demonstrated a state of withdrawal CNS hyperexcitability after a single hypnotic dose of ethanol (4 g/kg, p.o.). Details of the acute alcohol withdrawal procedure and HIC scoring system used in our work have been published (Metten et al., 1998; Kozell et al., 2008). Mice were scored twice for baseline (pre-ethanol) HICs 20 min apart, followed by a single hypnotic dose of ethanol (4 g/kg, i.p., in 20% w/v in saline) and then scored hourly between 2 and 12 h post-ethanol administration. To create an index of alcohol withdrawal independent of potential individual and/or genetic model differences in baseline HIC scores, post-ethanol HIC scores were corrected for individual baseline scores as in previous work (Kozell et al., 2008). Acute alcohol withdrawal severity was calculated as the area under the curve (i.e., the sum of the post-ethanol HIC scores) from 2 to 12 h post-ethanol.

#### Repeated alcohol model

Some animals were tested using an established repeated alcohol exposure paradigm (Chen et al., 2008). Animals were moved into a procedure room at least 1 h prior to beginning the experiment. Body weights were recorded before each ethanol injection. Baseline HICs were measured twice (20 min apart), immediately followed by a first dose of ethanol (4 g/kg) at 0 h, with alcohol administration repeated 8 and 20 h later, for a total of three doses. HIC testing began at 22 h and continued hourly through 32 h. Alcohol withdrawal severity was indexed as described above. Acute alcohol withdrawal severity was calculated as the area under the curve (i.e., the sum of the post-ethanol HIC scores) from 2 to 12 h post-ethanol.

### Anxiety-like behavior in the elevated zero maze (EZM) using alcohol withdrawn and control animals

The EZM apparatus and procedure used to assess anxiety-like behavior were based on previous studies (Kliethermes et al., 2004; Milner and Crabbe, 2008; Barkley-Levenson and Crabbe, 2015). The apparatus has an external diameter of 45 cm and consists of four proportional arms, two open and two closed, with a black acrylic floor (5.5 cm across) elevated 46 cm above the floor and placed in a large cob bedding filled tub to prevent potential fall-related injuries. Closed arm walls are 11 cm tall clear acrylic, with a small (3 mm) lip along the inner and outer edges of open arms to prevent falls. All testing occurred under dim lighting (15–20 lux) and was videotaped from above with camcorders. Mice were tested on two sets of two mazes concurrently with an opaque barrier between mazes, and mice were placed onto an open arm facing a closed arm at the start of a test. Before each subject was placed in the apparatus, the floor and walls were sprayed with 10% isopropanol and wiped with clean paper towels to eliminate odors.

#### Habituation

Mice were habituated to the apparatus for three days (prior to beginning ethanol dependence induction), and moved into the procedure room at least 1 h prior to the first habituation session. On each daily habituation, mice were removed from home cages and placed onto the open arm portion at the beginning of each 10 min session. Arm and placement remained the same throughout, and animals remained in the procedure room until transfer to inhalation chambers.

#### Alcohol dependence induction

Details of the chronic ethanol exposure method used to induce physical dependence have been published, and involve a standard paradigm in which adult mice are continuously exposed to ethanol vapor for 72 h (Terdal and Crabbe, 1994). Mice were weighed and scored twice (20 min apart) for baseline HICs prior to receiving either saline (air-control group) or a loading dose of ethanol (1.5 g/kg ethanol, i.p., 20% in saline). In addition, all mice received daily injections of pyrazole-hydrochloride (68 mg/kg, i.p.; alcohol dehydrogenase inhibitor) to stabilize blood and brain ethanol levels. Levels of ethanol in vapor (typically 6-8 mg ethanol/liter air) were selected to achieve approximately equal blood ethanol concentration (BEC) values across individuals and genetic models. After 24 and 48 h of ethanol vapor exposure, blood samples (20 μl) were drawn from 20 mice by tail nicking with a capillary tube, serving as an additional check on inhalation procedure efficacy in each pass and allowing minor adjustments to ethanol flow rates to maintain BEC values near the desired blood level (~1.5 mg/ml). At 72 h, all mice were removed from inhalation chambers. Blood samples were drawn from the ethanol-exposed mice for BEC analysis, and control animals were tail-nicked, but no blood was collected. Blood samples were analyzed soon after collection using headspace gas chromatography exactly as previously published (Finn et al., 2007).

#### EZM testing

Animals were moved into the procedure room at least 1 h prior to the testing session. Ethanol-dependent and control animals were tested in the EZM (10 min sessions) 24 and 48 h after removal from the chambers. Some genotypes (R2) were also tested 7 h after removal from chambers. Locomotor activity (distance traveled), time spent in open arms, and entries into arms were measured using Ethovision 8.5 XT video-tracking software (Noldus Information Technology, Inc.). Head dips in the same video clip were scored by an observer blinded to experimental treatment.

### Ethanol CPP

R3 congenic and WT animals (D2 genetic background) were tested using an established apparatus and paradigm (Cunningham, 2014). R8 congenic and WT animals (B6 genetic background) were tested using a slightly modified protocol (Tipps et al., 2015). CPP chambers (San Diego Instruments) are housed in illuminated, ventilated, and sound-attenuating chambers (AccuScan Instruments Inc) and consist of clear plastic walls 30 L × 15 W × 15 H cm equipped with exchangeable floor panels, which are themselves two textured interchangeable halves. The “grid” floor is constructed of 2.3 mm stainless steel rods mounted 6.4 mm apart, and the “hole” floor a stainless steel panel with 6.4 mm round holes aligned with 9.5 mm staggered centers. Horizontal activity and animal location are measured using photocell beam interruptions recorded by a fully automated, computer-connected system. The protocol involves three phases: habituation (1 session), conditioning (8–16 sessions) and testing (1–4 sessions). Animals were randomly assigned to conditioning groups and the groups counter-balanced. Chamber and floors were wiped down with a damp sponge after each animal. Sessions were conducted 5 days a week with a 2 day break between the first four and final four conditioning sessions for R3 congenic and WT littermate testing. Testing for R8 congenic and background strain animals was similar, but without breaks. Each animal was handled, weighed, and injected (i.p.) with saline (20 ml/kg) or ethanol (2 g/kg; 12.5% ethanol in saline) just before placement into the apparatus for each session. During the entire experiment the orientation of the floors remained the same for individual animals (i.e., if the grid floor was on the left side for habituation, it was on the left side for conditioning and test sessions).

On the habituation day, animals were injected with saline and placed immediately in CPP chambers with a hole floor on one side and grid floor on the other, with free access to both sides of the chamber. Habituation sessions lasted for 30 and 5 min, respectively, for R3 and R8 analyses. On four alternating days, animals were then conditioned using ethanol or saline during 5 min sessions with a single floor type (grid or hole), blocked by a clear acrylic divider from the other side of the chamber. R3 animals had 2 days off followed by another 4 conditioning sessions, for a total of 8 conditioning sessions (4 ethanol and 4 saline sessions). For R8 analyses, no breaks occurred between the sessions; instead, testing was performed after every 4 conditioning trials, for a total of 4 tests and 16 conditioning trials. Conditioning sessions were 5 min for R3 and WT littermates, and 15 min for R8 and WT animals. On the final test day, mice were injected with saline (no drug on board) and placed in the CPP apparatus for 30 min (R3 and WT) or 15 min (R8 and WT), with both floor types (grid and hole) available. Amount of time spent on the ethanol-paired floor (hole or grid) was the primary dependent variable measured.

### Two-bottle choice drinking

Two-bottle choice drinking was tested using a well-established paradigm (Phillips et al., 1994). All animals tested were acclimated to single housing in standard shoebox housing with paper bedding and a wire-top lid for at least 1 week prior to start of the experiment.

#### Ethanol consumption and preference

R8 congenic and WT animals received 24 h access to two 25 ml bottles containing tap water for 4 days, prior to exposure to 24 h access to ethanol (3, 6, 10, and 20%, 4 days each) or tap water. In a separate study, *Kcnj9*^−/−^ (B6 background) and WT littermates were assessed for ethanol consumption and preference using the same procedure. Ethanol consumption (expressed in g/kg/day) of each ethanol solution was calculated as the average of the 2nd and 4th day the solution was presented, and was analyzed as previously described (Phillips et al., 1994). Ethanol preference compared to tap water was calculated as volume ethanol/total fluid consumed in g/kg/day, as in our previous work (Milner et al., 2015).

#### Tastants (saccharin, quinine, and KCL)

Following a 4–5 day washout period (water exposure only), mice from the studies above were assessed for tastant consumption and preference. Animals had 24 h access to saccharin (0.033 and 0.066%), quinine (15 and 30 μM) and KCl (100 and 200 mM) for 4 days each. Consumption was calculated as the average of 2nd and 4th days of solution presentation, and expressed as mg/kg/day. Tastant preference compared to tap water was calculated as volume tastant/volume total fluid consumed in g/kg/day.

### Genotype analyses

DNA was extracted from ear punch tissue using the QuickExtract™ DNA Extraction Solution (Lucigen) according to manufacturer instructions. PCR amplification and gel electrophoresis was performed using SNP and simple sequence length polymorphism markers from the *D1Mit* series for mouse chromosome 1 (www.informatics.jax.org). *Kcnj9*^−/–^, *Kcnj9*^−/+^, and WT littermates were differentiated using a PCR-based assay with a common forward primer (G3com) and two reverse primers (G3WT and G3KO). Null mutant and wildtype animals produce 500 and 645 bp PCR products, respectively, and a heterozygote produces both. All PCR reactions are performed using Qiagen HotStar under standard conditions with a 55°C annealing temperature. The primer sequences are as follows: G3com (GATACTAGACTAGCGTAACTCTGGAT), G3WT (GATAAAGAGCACAGACTGGGTGTCG), G3KO (CAAAGCTGAGACATCTCTTTGGCTCTG).

### *Alcw1_1_* and *Alcw1_2_* candidate genes

Protein coding genes and non-coding RNAs within the maximal QTL interval were identified using Ensembl database for the reference B6 genome. (www.ensembl.org, GRCm38.p5). EMBL-EBI (https://www.ebi.ac.uk/gxa/home) was searched for evidence of brain expression in mouse or other species. Sequence variation was queried for non-synonymous coding region changes in any annotated transcript for each gene using MGI (Jackson Labs) database, specifically for B6 vs. D2 SNPs (single nucleotide polymorphisms). Gene expression analyses in our chromosome 1 congenics vs. background strain animals were from our previous publications of microarray and/or QPCR data (Denmark and Buck, 2008; Kozell et al., 2009; Walter et al., 2017).

### Data analyses

For analyses of normally distributed data (based on a nonsignificant Shapiro-Wilks test), we performed analysis of variance (ANOVA) followed by a post-hoc (Tukey) test. For comparisons in which data were not normally distributed, analyses using a non-parametric Kruskal-Wallis ANOVA on ranks, which generates a *U* statistic for two groups and an *H* statistic for more than 2 groups, followed by a post hoc Conover-Inman Test (Systat 13; Systat Systems, Inc.) were performed. Anxiety-like behavior was analyzed using an ANOVA followed by a one-tailed post-hoc Tukey's test, as in previous work showing heightened anxiety in alcohol-withdrawn animals compared to controls (Kliethermes et al., 2004). Ethanol CPP was assessed using a one sample *t*-test with the mean set to 0.5 (no preference). Data throughout are presented as the mean ± SEM, with significance (*p* < 0.05) indicated based on two-tailed analyses (unless one-tailed is specified). Percentage of total variance attributable to each R2 and R3 congenic strain for acute alcohol withdrawal was calculated based on *R*^2^-values from a one-way ANOVA by strain (SS_betweenstrains_/SS_total_) (Belknap et al., 1996).

## Results

### R3 congenic interval captures an alcohol withdrawal QTL on chromosome 1 (*Alcw1_2_*)

**Figure 1:**
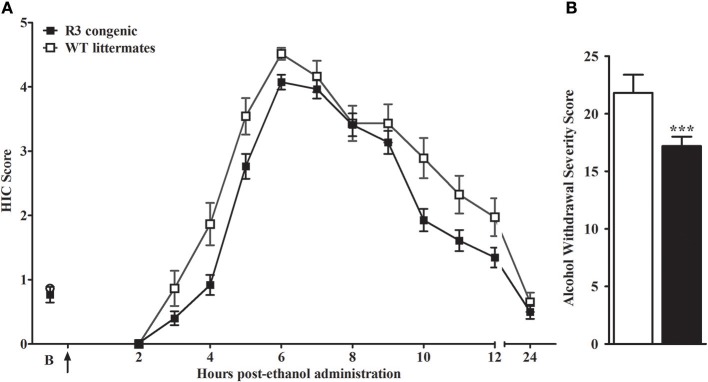
R3 congenic animals demonstrate a modest but significant reduction in alcohol withdrawal severity compared to WT animals. **(A)** HIC time course before and after ethanol administration (4 g/kg, i.p., indicated by the arrow) for R3 congenic and WT animals (*n* = 110 and 37, respectively). HICs were scored at baseline (“B”, i.e., pre-ethanol) and then hourly from 2 to 12 h post-ethanol and then again at 24 h. Baseline HIC scores did not differ between genotypes [*F*_(1, 143)_ = 0.1, *p* = 0.78, NS]. As ethanol is metabolized, HIC scores increase above baseline beginning about 4 h post-ethanol, indicating a state of withdrawal hyperexcitability, which peaks approximately 6–7 h post-ethanol exposure. **(B)** Alcohol withdrawal severity, calculated as the mean AUC12 ± SEM (from 2 to 12 h, and corrected for baseline scores), was significantly reduced in the R3 congenic compared to WT background strain animals (***p = 0.001).

We report the creation of a novel D2.B6 ISC model, R3. Genotypic analyses of R3 determined the minimal introgressed interval to be 7.2 Mb (164.30–171.35 Mb; maximal 164.17–171.36 Mb; build GRCm38). As shown in Figure 1, we tested for QTL capture by phenotypic comparisons of R3 congenic and WT animals using the same robust behavioral phenotype (acute alcohol withdrawal severity measured by the HIC) used to initially detect and confirm an alcohol withdrawal QTL to a large region of chromosome 1 (Buck et al., 1997). A main effect of sex is apparent (*p* = 1.5 × 10^−4^), but with no genotype x sex interaction (*p* > 0.2, NS), HIC data for both sexes were collapsed to increase statistical power of the analyses. A main effect of treatment was evident, with R3 congenics demonstrating significantly less severe withdrawal compared to background strain animals (withdrawal severity scores = 17.2 ± 0.8, and 21.8 ± 1.6, respectively; *F*_(1, 142)_ = 12.5, *p* = 0.001, Figure 1B). Our results confirm that a gene(s) affecting alcohol withdrawal is captured within the R3 introgressed interval.

### Comparison of R3 and R2 ISCs delineates a second QTL with a larger effect size on risk for alcohol withdrawal (*Alcw1_1_*)

**Figure 2:**
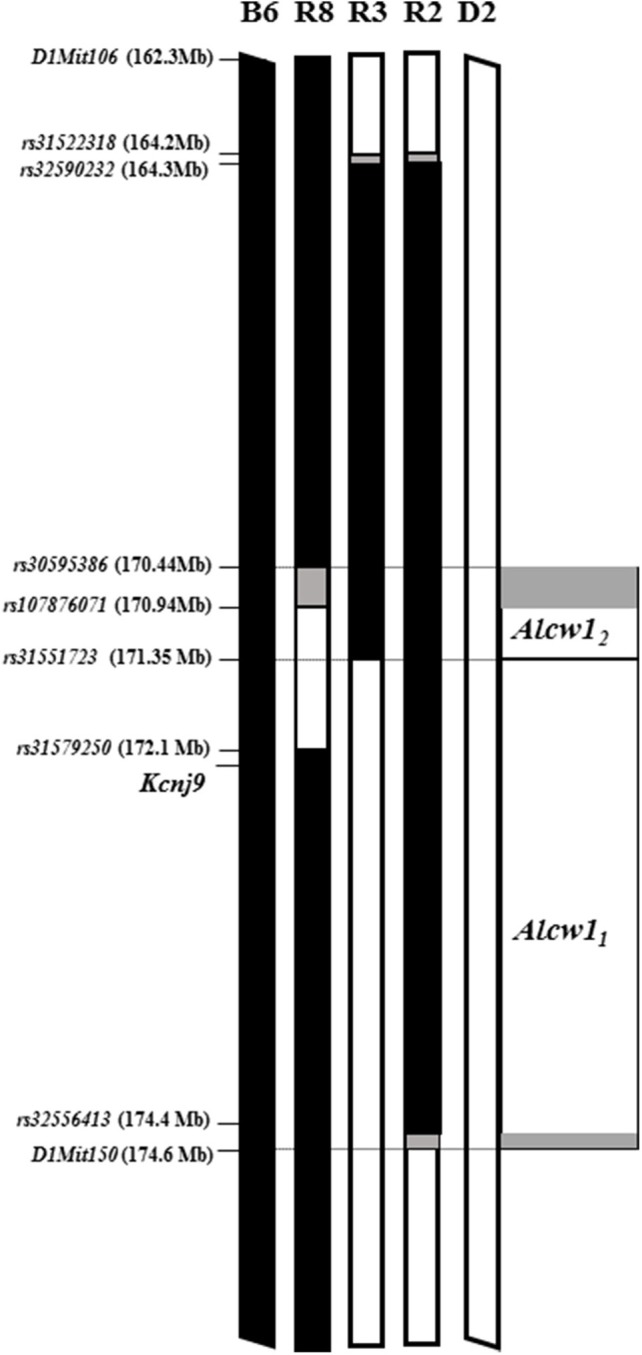
*Alcw1*_1_ and *Alcw1*_2_ localization using multiple, reciprocal ISC genetic models. Allelic status at genetic markers across the region of chromosome 1 that spans *Alcw1*_1_ and *Alcw1*_2_ is illustrated for the two progenitor strains (B6 and D2), two D2.B6 congenic strains (R3; and R2, Walter et al., 2017), and one B6.D2 congenic strain (R8; Kozell et al., 2008). The genetic markers used to establish the introgressed interval boundaries are indicated, with their locations also given. Chromosomal regions homozygous for the B6 strain allele are shown in black. Chromosomal regions homozygous for the D2 strain allele are shown in white. The boundary regions, within which the precise transition exists but is not currently known, are shown in gray. In some cases the boundary region is so small that it is not visible. The *Alcw1*_2_ interval as shown is defined as donor region that is common to the reciprocal R8 and R3 congenic strains. The *Alcw1*_1_ interval as shown is defined as that region of the R2 congenic introgressed interval that excludes the *Alcw1*_2_ interval.

We recently created a second D2.B6 ISC (R2) with a larger introgressed interval (minimal 10.2 Mb, 164.3–174.5 Mb; maximal 164.1–174.6 Mb; Build GRCm38; Walter et al., 2017), which spans entirely and extends beyond the R3 introgressed interval (Figure 2). Our data clearly show that the effect size accounted for in the R2 congenic is significantly greater than that accounted for by R3, contributing 16 and 3%, respectively, of the genetic variance in acute alcohol withdrawal severity. Taken together, our results confirm the existence of an alcohol withdrawal QTL within the R3 introgressed interval (contributing 3% of the genetic variance), and point to the existence of an additional alcohol withdrawal QTL also within the larger R2 introgressed interval (which, by subtraction, we estimate contributes 13% of the genetic variance). Given the larger effect size of the latter, these two QTLs are termed *Alcw1*_2_ and *Alcw1*_1_, respectively. The minimal *Alcw1*_1_ interval is 3.11 Mb (171.37–174.47 MB), and maximal 3.28 Mb (171.35–174.63 Mb), which includes proximal (0.02 Mb) and distal (0.16 Mb) boundary regions.

### Comparison of R3 and R8 ISC models suggest more precise localization of *Alcw1_2_*

Our previous analyses comparing R8 congenic and background strain (B6) animals confirmed capture of a locus/loci affecting alcohol withdrawal severity using both acute and chronic models, and contributes 6% of the genetic variance in acute alcohol withdrawal severity (Kozell et al., 2008). As illustrated in Figure 2, the small R8 introgressed interval (1.2–1.7 Mb; minimal 170.9–172.1 Mb, maximal 170.4–172.1 Mb; Build GRCm38p5; Kozell et al., 2008) largely overlaps that of R3. Furthermore, given the comparable QTL effect size accounted for by these two congenic models (6 and 3%, respectively), *Alcw1*_2_is likely captured in both R8 and R3. If so, *Alcw1*_2_ would now be localized to a very narrow 405-923 Kb interval (minimal 170.94–171.35 Mb, maximal 170.44–171.37 Mb). Arguably even more importantly, the creation of R3 and R8 *Alcw1*_2_ congenic models with different inbred D2 and B6 genetic backgrounds, respectively, are invaluable genetic tools to begin to test potential pleiotropic *Alcw1*_2_ effects on diverse phenotypes, including behavioral tests limited by genetic background (below).

### *Alcw1_1_* and *Alcw1_2_* interval resident genes with validated expression in the brain

The R2 congenic introgressed interval spans the R3 interval and extends distally another 3.28 Mb, (maximal interval; See Figure 2). *Alcw1*_1_ is defined as that part of the R2 interval that does *not* overlap with the R3 interval. The minimal *Alcw1*_1_ interval contains 77 protein coding genes and an additional 3 coding genes lie within the maximal boundaries. Within the maximal interval, there are also 29 pseudogenes 10 long noncoding RNAs, and 9 short noncoding RNAs annotated. A total of 48 coding genes have confirmed expression in the brain and are presented in Table 1. Twenty-five have at least one B6 vs. D2 nonsynonymous coding SNP, and 20 have evidence of differential mRNA expression between congenic and background strain mice, indicating *cis*-regulation.

**Table 1:** *Alcw1*_1_ interval protein coding genes.

**Congenic vs. WT**		
**Gene**	**ΔSeq**	**ΔExp**
*Pfdn2*	0	+
*Klhdc9*	2	+
*Nectin4*	0	nd
*Arhgap30*	5	–
*Usf1*	0	+
*Tstd1*	0	+
*F11r*	0	–
*Alyref2*	3	nd
*Cd244*	14	–
*Slamf7*	4	+
*Cd48*	3	–
*Slamf1*	0	–
*Cd84*	2	–
*Gm10521*	0	nd
*Slamf6*	1	–
*Vangl2*	1	+
*Nhlh1*	0	–
*Ncstn*	3	+
*Copa*	2	+
*Pex19*	2	+
*Dcaf8*	0	+
*Pea15a*	0	–
*Casq1*	0	+
*Atp1a4*	4	+
*Igsf8*	2	+
*Atp1a2*	0	–
*Kcnj9*	0	+
*Kcnj10*	5	+
*Pigm*	1	–
*Slamf9*	3	+
*Igsf9*	4	+
*Tagln2*	0	–
*Cfap45*	0	–
*Vsig8*	2	+
*Slamf8*	3	–
*Fcrl6*	6	nd
*Dusp23*	0	–
*Crp*	1	–
*Apcs*	0	–
*Fcer1a*	2	–
*Ackr1*	0	nd
*Cadm3*	1	+
*Aim2*	0	–
*Pydc3*	0	nd
*Mnda*	0	nd
*Ifi203*	0	–
*Spta1*	0	nd
*Fmn2*	2	+

*The Alcw1_1_ interval protein coding genes with demonstrated expression in whole brain are listed in order of location, from most proximal to most distal, including those within the minimal distal boundary region (gray shaded; Figure 2). The number of validated non-synonymous SNPs (ΔSeq) between the two progenitor strains are indicated. Genes showing significant differential mRNA expression (ΔExp) between congenic and background strain animals are indicated (+), as is the absence of detected DE (-); (nd) indicates no current data comparing chromosome 1 congenic and background strain animals. Other protein coding genes not shown include olfactory receptor genes, interferon activated genes, and four genes (Ly9, Mptx2, Mptx1 and Mnda1) which lack evidence of brain expression. Additionally, there are many pseudogenes and noncoding RNAs within this Alcw1_1_ interval that are not shown*.

The *Alcw1*_2_ interval as shown in Figure 2 is defined as donor region that is common to the reciprocal R8 and R3 congenic strains. The minimal region contains 19 protein coding genes with an additional 9 in the boundary regions. All of these exhibit some evidence of brain expression and are listed in Table 2. Thirteen have at least one B6 vs. D2 nonsynonymous coding SNP, and 15 have evidence of differential mRNA expression between congenic and background strain mice, indicating *cis*-regulation. Within the maximal interval, there are also 5 pseudogenes, 4 long noncoding RNAs, and 7 short noncoding RNAs annotated. Two coding genes (*Pfdn2* and *Klhdc9*) are within the shared boundary region (i.e., *Alcw1*_2_ distal boundary and proximal *Alcw1*_1_ boundary).

**Table 2:** *Alcw1*_2_ interval protein coding genes.

**Congenic vs. WT**		
**Gene**	**ΔSeq**	**ΔExp**
*Nos1ap*	0	+
*Olfml2b*	0	nd
*Atf6*	1	+
*Dusp12*	3	–
*Gm26620*	0	nd
*Fcr1b*	0	nd
*Fcrla*	0	nd
*Fcgr2b*	0	–
*Fcgr4*	0	–
*Fcgr3*	4	+
*Cfap126*	2	–
*Sdhc*	3	+
*Mpz*	1	–
*Pcp4l1*	0	nd
*Nrli3*	1	–
*Tomm40l*	0	+
*Apoa2*	0	+
*Fcer1g*	5	–
*Ndufs2*	1	+
*Adamts4*	2	+
*B4galt3*	0	+
*Ppox*	0	+
*Usp21*	1	+
*Ufc1*	0	+
*Dedd*	0	–
*Nit1*	1	+
*Pfdn2*	0	+
*Klhdc9*	2	+

*The Alcw1_2_ interval protein coding genes with demonstrated expression in whole brain are listed in order of location, from most proximal to most distal, including those within the minimal distal boundary region (grey shaded; Figure 2). The number of validated non-synonymous SNPs (ΔSeq) between the two progenitor strains are indicated. Genes showing significant differential mRNA expression (ΔExp) between congenic and background strain animals are indicated (+), as is the absence of detected DE (-); (nd) indicates no current data comparing chromosome 1 congenic and background strain animals. Additionally, there are many pseudogenes and noncoding RNAs within the Alcw1_2_ interval that are not included in the table*.

### Anxiety-like behavior in *Alcw1_1_* and *Alcw1_2_* congenic models in alcohol dependent and control animals

To begin to assess the potential broader effects of *Alcw1*_1_ beyond alcohol withdrawal enhanced HICs, we tested ISC (R3 and R2) and appropriate WT background strain animals for withdrawal-induced anxiety-like behavior in the EZM. We initially employed the acute alcohol withdrawal paradigm, but were unable to reliably detect any anxiety-like behavior using the B6.D2 R8 congenic (not shown). Thus, we report here results for the chronic alcohol withdrawal protocol in which animals are rendered alcohol-dependent by continuous (72 h) exposure to ethanol vapor in an inhalation chamber and compared to appropriate controls (adjacent air control chambers). R3 congenic and WT littermates were tested 24 and 48 h after removal from chambers. R2 congenic and WT littermates were tested at 7 (see Supplementary Figure 1), 24 and 48 h after removal from the chambers. Main effects of treatment (ethanol withdrawn vs. air control) and genotype (R2 vs. WT, R3 vs. WT), as well as potential genotype X treatment (GXT) interactions were assessed (below).Genotype-dependent differences in BEC values were not detected after 24, 48, or 72 h continuous ethanol vapor exposure (Supplementary Table 1; all *p* > ~0.3, NS). We applied an EZM habituation procedure shown to be crucial to detecting alcohol withdrawal associated anxiety-like behavior in dependent mice (Kliethermes et al., 2004): all animals were placed in the EZM apparatus for 10 min on three sequential days of the week prior to vapor chamber testing. Across habituation days, we observed significant decreases in distance traveled, time spent in open arms, open arm entries and head dips within subjects (Supplementary Tables 2, 3; *p* < 0.05), and a main effect of genotype on distance traveled in R2 vs. WT [*F*_(2, 43)_ = 10.3, *p* = 0.003], with R2 traveling less distance than WT littermates. However, no treatment or GXT interactions (all *p* > 0.7 and *p* > 0.2, respectively, NS) were detected. Because no differences due to treatment nor GXT interactions were apparent during habituation days, differences in activity and measures of anxiety detected post-ethanol exposure are not likely explained by potential strain-dependent apparatus habituation.

#### Percent time in the open arms

**Figure 3:**
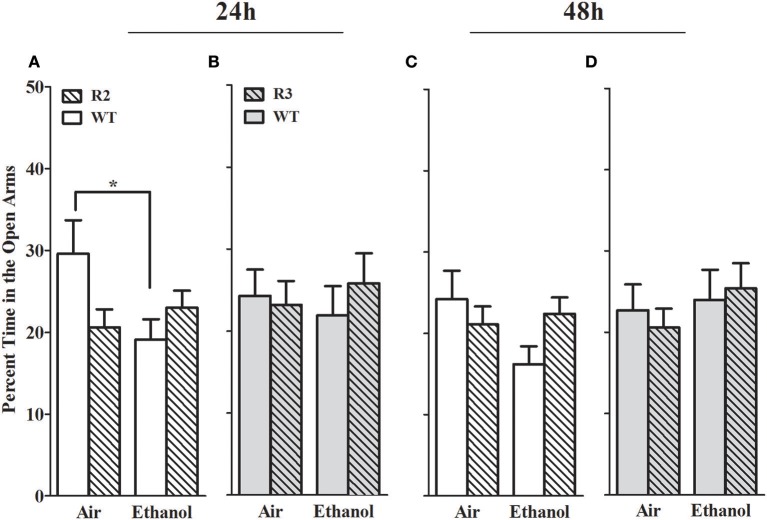
Percent time in the open arms of the EZM in withdrawn (24 and 48 h after cessation of chronic ethanol exposure) and control (air-pyrazole) animals: R2 congenic vs. WT and R3 congenic vs. WT comparisons. Panels show the percentage of time (mean ± SEM) animals spent in the open arm of the EZM during a 10 min test. **(A)** R2 vs. WT (24 h): No main effects of genotype (*p* = 0.37) or treatment (*p* = 0.18) are detected. Nonetheless, there was a significant GXT interaction (*p* < 0.03), with withdrawn WT (but not R2 congenic) animals spending less time in the open arms compared to air-control animals (*p = 0.028, one-tailed). **(B)** R3 vs. WT (24 h): No main effects of treatment, genotype, or GXT interaction were detected (all *p* > 0.5). **(C)** R2 vs. WT (48 h): No main effects of treatment (*p* = 0.4), genotype (*p* = 0.2, NS) or GXT interaction is apparent (*p* = 0.17). **(D)** R3 vs. WT (48 h): there were no effects of ethanol withdrawal on percent open arm time in the R3 mice compared to WT littermate for treatment, genotype, or GXT interaction (all *p* > 0.5). *p < 0.05, GXT *post hoc* analysis, withdrawn significantly different from air-controls.

The percent time spent in the open arms is a well-established measurement of anxiety-like behavior in the EZM (Milner and Crabbe, 2008; Barkley-Levenson and Crabbe, 2015), and for which ethanol withdrawal-induced anxiety-like behavior has been observed (Kliethermes et al., 2004). As shown in Figure 3, no main effect of treatment (all *p* > 0.18; Figures 3A,C) genotype were detected (all *p* > 0.37, NS). A significant GXT interaction was evident at 24 h [*F*_(1, 42)_ = 5.2, *p* = 0.028], but not 48 h [*F*_(1, 42)_ = 1.9, *p* = 0.17] post-ethanol. Ethanol withdrawn WT animals spent less time in the open arms compared to air-control WT at 24 h post-ethanol (*p* = 0.028, 1-tailed). Ethanol withdrawn R2 animals did not differ from air-control animals in time spent in the open arms at 24 or 48 h (both *p* > 0.8, NS). Although R2 and WT air control animals did not differ in percent time spent in the open arm at 24 h (*p* = 0.23) or 48 h (*p* = 1.0), it is possible that these non-significant differences may also contribute to the significant GXT interactions identified. Overall, these results are consistent with the conclusion that WT animals show more robust and longer lasting withdrawal-induced anxiety like behavior than R2 congenic animals.

In contrast, in the R3 congenic and WT analyses, no main effect of treatment was detected at either time point assessed (24 and 48 h post-ethanol, both *p* > 0.5, Figures 3B,D). No main effect of genotype detected at either time point tested (both *p* > 0.6, NS), and no GXT interactions detected (both *p* > 0.5, NS). Taken together, these results suggest that a gene(s) within the R2 interval significantly affects alcohol withdrawal-induced anxiety-like behavior (and with the same direction of effect as for alcohol withdrawal enhanced HIC severity).

#### Number of open arm entries

**Figure 4:**
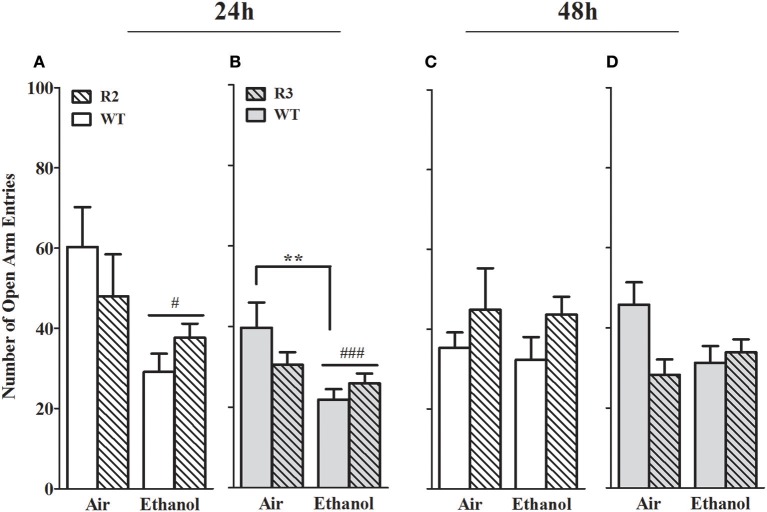
Number of open arm entries in the EZM using withdrawn (24 and 48 h after cessation of chronic ethanol exposure) and air-control animals: comparison of R2 congenic and WT analyses and R3 congenic and WT analyses. These data represent the number of entrances into the open arms (mean ± SEM) during the 10 min test. **(A)** R2 vs. WT (24 h): a main effect of treatment is evident (*p* = 0.002) but no main effect of genotype (*p* = 0.77). A trend for a GXT interaction is detected (*p* = 0.055, one-tailed). **(B)** R3 vs. WT (24 h): a significant main effect of treatment is apparent (*p* = 0.002) but no main effect of genotype (*p* = 0.41). However, a trend for a GXT interaction is detected (*p* = 0.035, one-tailed), with WT withdrawn mice making fewer entries into open arms than air-exposed mice (**p = 0.007). **(C)** R2 vs. WT (48 h): no main effects of treatment (*p* = 0.62) or genotype (*p* = 0.31), or GXT interaction (*p* = 0.29) were detected. **(D)** R3 vs. WT (48 h): no main effect of treatment is evident (*p* = 0.30). A trend for a main effect of genotype is detected (*p* = 0.078), and a significant GXT interaction (*p* = 0.02), with ethanol-withdrawn WT littermates showing a trend to spend less time in the open arms than air-control animals (*p* = 0.055, one-tailed). ^#^*p* < 0.05, ^###^*p* < 0.001, main effect of treatment. *p < 0.05, GXT *post hoc* analysis, withdrawn significantly different from air-controls.

**Figure 5:**
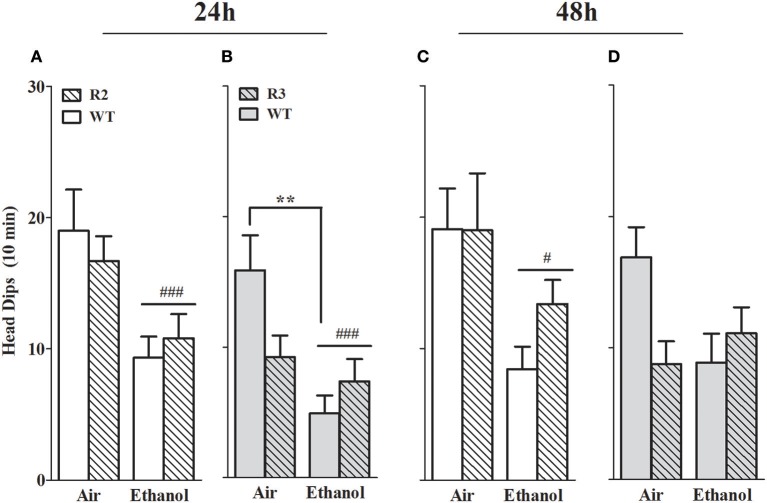
Head dips measured in the EZM using ethanol withdrawn (24 and 48 h post-ethanol) and control animals: R2 congenic vs. WT and R3 congenic vs. WT comparisons. Panels show the number of head dips over the side of the open arms of the EZM apparatus during a 10 min test. **(A)** R2 and WT (24 h): ethanol withdrawn mice made fewer head dips than air-controls (*p* = 0.001). No main effect of genotype (*p* = 0.27) nor GXT interaction (*p* = 0.4) were detected. **(B)** R3 and WT (24 h): no main effects due to genotype (*p* = 0.27) were detected. Both a main effect of treatment (*p* = 0.001) and a GXT interaction (*p* = 0.02) are evident, with ethanol-withdrawn WT littermates making significantly fewer head dips than their air-controls (**p = 0.002*)*. **(C)** R2 and WT (48 h): no main effect of genotype (*p* = 0.35) or GXT interaction (*p* = 0.33) were detected. However, a main effect of treatment is apparent (*p* = 0.003). **(D)** R3 and WT (48 h): no main effects of genotype (*p* = 0.24) or treatment (*p* = 0.17) were detected. A significant GXT interaction is apparent (*p* = 0.033), with withdrawn WT showing a trend for fewer head dips compared to WT air-control animals (*p* = 0.055, one-tailed). ^#^*p* < 0.05; ^###^*p* < 0.001, main effect of treatment. **p < 0.01, GXT *post hoc* analysis, withdrawn significantly different from air-controls.

The number of open arm entries is another well-established measurement of anxiety-like behavior in the EZM (Milner and Crabbe, 2008; Barkley-Levenson and Crabbe, 2015), and for which ethanol withdrawal-induced anxiety-like behavior has been observed (Kliethermes et al., 2004). As shown in **Figure 5**, a main effect of treatment was evident at the 24 h withdrawal time point, with ethanol withdrawn R2 congenic and WT animals exhibiting a robust reduction in open arm entries [*F*_(1, 41)_ = 10.6, *p* = 0.002, Figure 4A], but not maintained 48 h post-ethanol [*F*_(1, 42)_ = 0.25, *p* > 0.6, Figure 4C]. No main effect of genotype was detected (*p* > 0.3). A trend for a GXT interaction was detected at 24 h [*F*_(1, 41)_ = 2.7, *p* = 0.055, one-tailed], with ethanol withdrawn R2 animals not differing from air-control R2 animals (*p* > 0.6, NS), while ethanol withdrawn WT animals made fewer entrances into the open arms compared to air-control WT animals (*p* = 0.007). There were no GXT interactions at 48 h [*F*_(1, 42)_ = 1.1, *p* = 0.29; Figure 4C] post-ethanol.

In the R3 and WT comparison, a significant main effect of treatment was evident 24 h post-ethanol, with ethanol withdrawn R3 and WT animals exhibiting a robust reduction in open arm entries compared to air-control animals [*F*_(1, 50)_ = 10.8, *p* = 0.002, Figure 4B]; but was not maintained 48 h post-ethanol [*F*_(1, 50)_ = 1.1, *p* > 0.29, NS, Figure 4D]. No main effect of genotype was detected (both *p* > 0.27, NS). However, a significant GXT interaction was apparent 24 h post-ethanol [*F*_(1, 50)_ = 3.7, *p* = 0.03, 1-tailed] comparison, with ethanol withdrawn WT littermates making fewer entrances into the open arms compared to their air-controls (*p* = 0.006), while ethanol withdrawn R3 congenic animals did not differ from their air-controls (*p* > 0.7, NS). Finally, there was a GXT significant interaction at 48 h [*F*_(1, 50)_ = 3.7, *p* = 0.048, 1-tailed] but there were no differences between ethanol withdrawn R3 or WT and their respective air-controls (both *p* > 0.29; NS). These results indicate that a gene(s) within the R3 introgressed interval has a significant effect on alcohol withdrawal-induced anxiety-like behavior (and with the same direction of effect as for alcohol withdrawal enhanced HIC severity). Given the modest effect size in the R3 vs. WT analyses compared to the R2 vs. WT analyses, our results may also suggest the influence of a second locus within the R2 interval (distinct from the R3 interval) that also affects alcohol withdrawal-induced anxiety-like behavior (again, with the same direction of effect as for alcohol withdrawal enhanced HIC severity). Although R3 and WT air control animals did not differ in open arm entries at 24 h (*p* = 0.32) or 48 h (*p* = 0.30), it is possible that these non-significant differences may also contribute to the significant GXT interactions found.

#### Head dips

**Figure 6:**
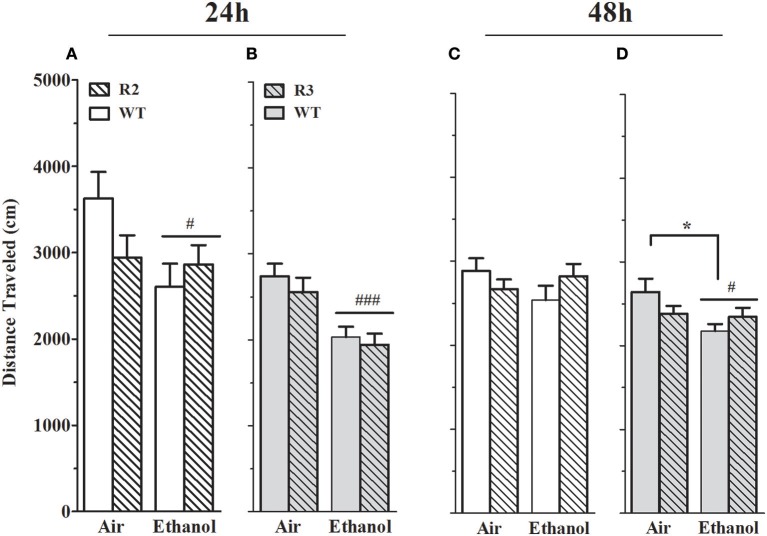
Distance traveled on EZM is reduced in alcohol withdrawn R2, R3 and WT mice compared to air-control animals. Panels show the total distance traveled (mean ± SEM) on the EZM during a 10 min test. **(A)** R2 vs. WT (24 h): a main effect of treatment (*p* = 0.03, one-tailed) is apparent, with alcohol withdrawn mice traveling less distance than air-controls. Although there is no main effect of genotype (*p* = 0.4), a trend for a GXT interaction is detected (*p* = 0.06, one-tailed). **(B)** R3 vs. WT (24 h): a main effect of treatment is evident, with ethanol withdrawn animals traveling significantly less distance than the air-controls (*p* < 4x10^−6^). There is no main effect of genotype (*p* = 0.4) or GXT interaction (*p* = 0.8). **(C)** R2 vs. WT (48 h): no main effects of treatment, genotype, or interaction are detected (all *p* > 0.1). (**D**) R3 vs. WT (48 h): a main effect of treatment (*p* = 0.02), with ethanol withdrawn animals traveling significantly less distance than air-controls. There is also a significant GXT interaction (*p* = 0.03), with ethanol withdrawn WT (but not R3) animals moving significantly less than their air-control group (*p = 0.02). ^#^*p* < 0.05, ^###^ *p* < 0.001, main effect of treatment; *p < 0.05, GXT *post hoc* analysis, withdrawn significantly different from air-control group.

Head dips over the side of the apparatus are a measurement of exploratory behavior, and furthermore, animals exhibiting anxiety-like behavior are also significantly less likely to scan over the side of the apparatus and thus demonstrate fewer head dips than control animals (Weiss et al., 1998; Morgan et al., 2018). We therefore also measured head dips in the open arms. In the R2 congenic and WT analyses, a main effect of treatment was evident at all the withdrawal time points tested, with ethanol withdrawn R2 and WT animals exhibiting a robust reduction in head dips compared to air-controls at 24 h, [*F*_(1, 41)_ = 12.5, *p* = 0.001; Figure 5A] and 48 h post-ethanol [*F*_(1, 43)_ = 9.9, *p* = 0.003; Figure 5C]. No main effect of genotype was detected (all *p* > 0.27). No GXT interaction was detected at 24 h post-ethanol [*F*_(1, 41)_ = 0.73, *p* = 0.40; Figure 6A] or 48 h [*F*_(1, 43)_ = 1.0, *p* = 0.33; Figure 5C].

In the R3 and WT analyses, a robust main effect of treatment was evident 24 h [*F*_(1, 50)_ = 11.4, *p* = 0.001; Figure 5B] but not 48 h [*F*_(1, 50)_ = 1.6, *p* = 0.17; Figure 5D] post-ethanol. Although no main effect of genotype was detected (all *p* > 0.24), significant GXT interactions were apparent both 24 h [*F*_(1, 50)_ = 5.8, *p* = 0.02; Figure 5B] and 48 h [*F*_(1, 51)_ = 4.8, *p* = 0.033; Figure 5D] post-ethanol. At 24 h ethanol withdrawn WT animals made significantly fewer head dips than their air controls (*p* = 0.002) while at 48 h there was a trend for fewer head dips in the ethanol withdrawn WT compared to air controls (*p* = 0.11). These results indicate that a gene(s) within the R3 introgressed interval significantly affects alcohol withdrawal-induced exploratory/anxiety-like behavior. Here, R3 and WT air control animals show a trend for a difference in the number of head dips at 24 h (*p* = 0.12) and 48 h (*p* = 0.15), so it is possible that these non-significant differences may contribute to the significant GXT interaction identified. However, R2 and WT air-controls exhibit comparable numbers of head dips and demonstrate a significant GXT interaction, suggesting that a difference between the congenic and WT air-control groups is not required to observe a significant GXT interaction.

#### Distance traveled

To appropriately interpret EZM results, important primary and control behaviors were assessed, including total distance traveled. *Furthermore, alcohol withdrawal has been shown to be associated with reduced activity in the EZM* (Kliethermes et al., 2004), *and may represent an additional measure of alcohol withdrawal. However, it should be kept in mind that the extent to which this phenotype may (or not) be centrally mediated is not known*. As shown in Figures 6A,C, a main effect of treatment was evident: ethanol-withdrawn R2 and WT animals show less distance traveled compared to control (air) animals at 24 h [*F*_(1, 42)_ = 3.7, *p* = 0.03, one-tailed] post-ethanol, though not at 48 h [*F*_(1, 43)_ = 0.34, *p* = 0.5, NS]. No main effect of genotype was detected (all *p* > 0.4), but trends for a GXT interaction were detected at 24 h [*F*_(1, 42)_ = 2.7, *p* = 0.11] and 48 h [*F*_(1, 43)_ = 2.3, *p* = 0.14].

As shown in Figures 6B,D, a main effect of treatment was also evident using R3 and WT animals, with ethanol-withdrawn animals exhibiting a significant reduction in distance traveled both 24 and 48 h post-ethanol [*F*_(1, 51)_ = 20.4, *p* < 5 × 10^−5^, and *F*_(1, 51)_ = 4.7, *p* = 0.002, respectively]. No main effect of genotype (all *p* > 0.3), nor GXT interaction at 24 h (*p* > 0.75) was detected. However, a small but significant GXT interaction was apparent between R3 and WT [*F*_(1, 51)_ = 4.8, *p* = 0.03, Figure 6D], with a slight withdrawal-induced reduction in distance traveled still evident in WT littermates 48 h post-ethanol (*p* = 0.02), but no longer detected in R3 animals (*p* = 0.99). Importantly, this GXT interaction between R3 and WT littermates indicates that WT animals are affected by this alcohol withdrawal symptom to a greater extent than *Alcw1*_2_ congenic animals, consistent with the direction of effect for alcohol withdrawal enhanced HICs).

### *Kcnj9*^−/−^ (D2 genetic background) animals demonstrate significantly less severe acute alcohol withdrawal than WT littermates

**Figure 7:**
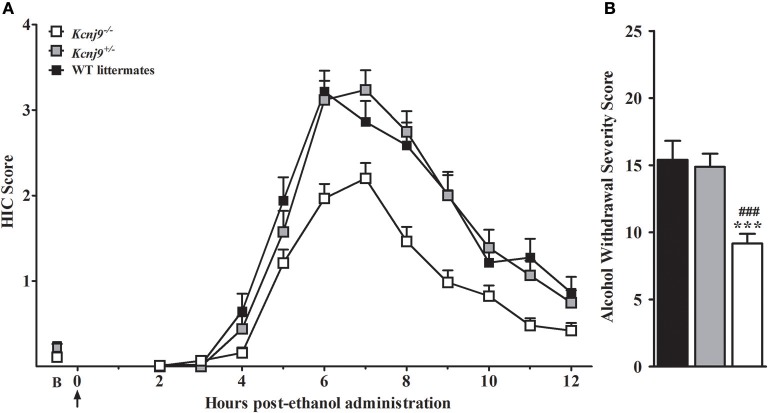
*Kcnj9*^−/−^ null mutant homozygotes (D2 background) demonstrate less severe alcohol withdrawal convulsions (acute model) than *Kcnj9*^+/−^ heterozygote and WT littermates. **(A)** HIC time course before and after ethanol administration (4 g/kg, i.p., indicated by the arrow) using *Kcnj9*^−/−^, *Kcnj9*^+/−^ and WT littermates (*n* = 119, 59 and 51, respectively). HICs were scored at baseline (“B”, i.e., pre-ethanol) and then hourly from 2 to 12 h post-ethanol. Baseline HIC scores did not differ among genotypes [*F*_(2, 144)_ = 1.2, *p* = 0.3]. As ethanol is metabolized, HIC scores increase above baseline, indicating a state of withdrawal hyperexcitability, which peaks approximately 6–7 h post-ethanol exposure. **(B)** Alcohol withdrawal severity, which was calculated as the AUC ± SEM from 2 to 12 h (corrected for baseline scores), and was significantly different among genotypes (*p* < 4 × 10^−7^). *Post hoc* analysis indicated that ethanol withdrawal severity was attenuated in *Kcnj9*^−/−^ compared to *Kcnj9*^+/−^ and WT littermates (###*p* = 8.1 × 10^−6^ and ***p = 1.5 × 10^−5^, respectively).

Our previous work identified a QTL for pentobarbital withdrawal (*Pbw1*, Buck et al., 1999), with our subsequent work precisely localizing *Pbw1* (Kozell et al., 2009) to a region within chromosomal region *Alcw1*_1_ (Figure 2) and identify *Kcnj9* as a high quality candidate gene (QTG) to underlie its phenotypic effects. Here, we report our results using two *Kcnj9*^−/−^ knockout models (with two different, inbred genetic backgrounds). Because no main effect of sex (*p* > 0.15) or sex × genotype interaction (SXG; *p* > 0.20; *n* = 17 to 22 sex/genotype) were detected, the data for both sexes were combined for the subsequent analyses. As shown in Figure 7, using D2.*Kcnj9*^−/−^ model previous created by us (Kozell et al., 2009), a significant main effect of genotype is apparent [*H*_(2, 153)_ = 28.7, *p* < 6 × 10^−7^, *n* = 119, 59 and 51, respectively], with alcohol withdrawal was less severe in *Kcnj9*^−/−^ compared to both D2-*Kcnj*^+/−^ and WT littermates (*p* = 7 × 10^−7^ and *p* = 3 × 10^−5^ respectively, Figure 7B).

### *Kcnj9*^−/−^ (B6 genetic background) mice demonstrate reduced withdrawal severity compared to WT littermates using a repeated alcohol withdrawal paradigm

**Figure 8:**
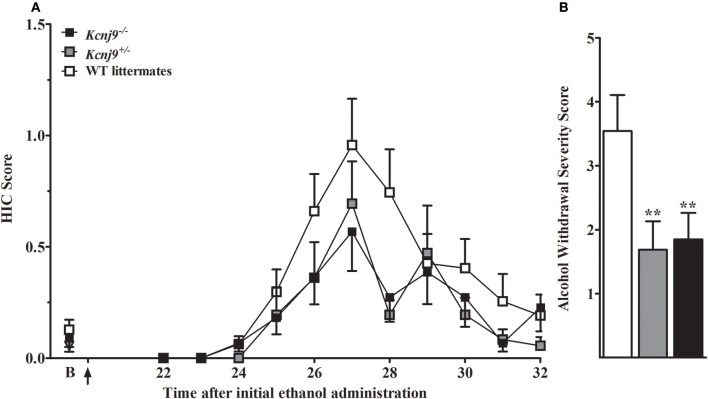
*Kcnj9*^−/−^ null mutant homozygotes (B6 background) show less severe alcohol withdrawal (repeated ethanol model) than WT littermates. **(A)** HIC time course before and after ethanol administration (4 g/kg, i.p., alcohol administered at 0, 8, and 20 h). The last of three ethanol injections is indicated by arrow (at 20 h). Baseline HICs did not differ among genotypes [*F*_(2, 124)_ = 0.8, *p* = 0.4]. HICs were scored at baseline (indicated by “B”) and hourly from 22 h until 32 h. As alcohol is metabolized, HIC scores increase above baseline, indicating a state of withdrawal hyperexcitability. **(B)** Repeated episodes of alcohol intoxication and withdrawal significantly enhanced alcohol withdrawal severity, which was indexed as the AUC ± SEM (corrected for baseline scores). There was a significant difference in ethanol withdrawal severity among genotypes (*p* = 0.008; *n* = 43, 35 and 48, respectively). *Post hoc* analyses indicated that *Kcnj9*^−/^^−^ or *Kcnj9*^+/−^ had significantly less severe alcohol withdrawal compared WT littermates (**p = 0.009 and **p = 0.006, respectively, compared to WT littermates).

We initially employed the acute alcohol withdrawal model, but were unable reliably detect withdrawal enhanced HIC severity above baseline scores using B6 background *Kcnj9*^−/–^ or WT genetic models (not shown), and thus did not replicate results of Herman et al. (2015) who reported a significant genotype effect at 8 h post-ethanol using male B6 background *Kcnj9*^−/−^ mice. Therefore, the present studies use mice tested using a repeated alcohol withdrawal model which can yield more robust withdrawal (Chen et al., 2008). B6 background *Kcnj9*^−/–^, *Kcnj9*^−/+^ and WT received three doses of ethanol (4 g/kg), at 0, 8 and 20 h, as this has previously been shown to result in enhanced withdrawal HICs in B6 strain and B6-derived genetic models (Chen et al., 2008), followed by HIC scoring from 22 to 32 h (Figure 8A). Because no main effect of sex (*p* > 0.6,) or SXG interaction (*p* > 0.9; n = 16–26 sex/genotype) were detected, the data for both sexes were combined for the subsequent analyses. We observed a robust main effect of genotype on ethanol withdrawal severity [*H*_(2, 128)_ = 9.6, *p* = 0.008; *n* = 43, 35 and 48, respectively], with *Kcnj9*^−/−^ and *Kcnj9*^+/−^ both demonstrating significantly less severe withdrawal than WT littermates (*p* = 0.009 and *p* = 0.006, respectively; Figure 8B).

### Ethanol drinking and preference in *Alcw1_2_* congenic (R8) and WT animals

**Figure 9:**
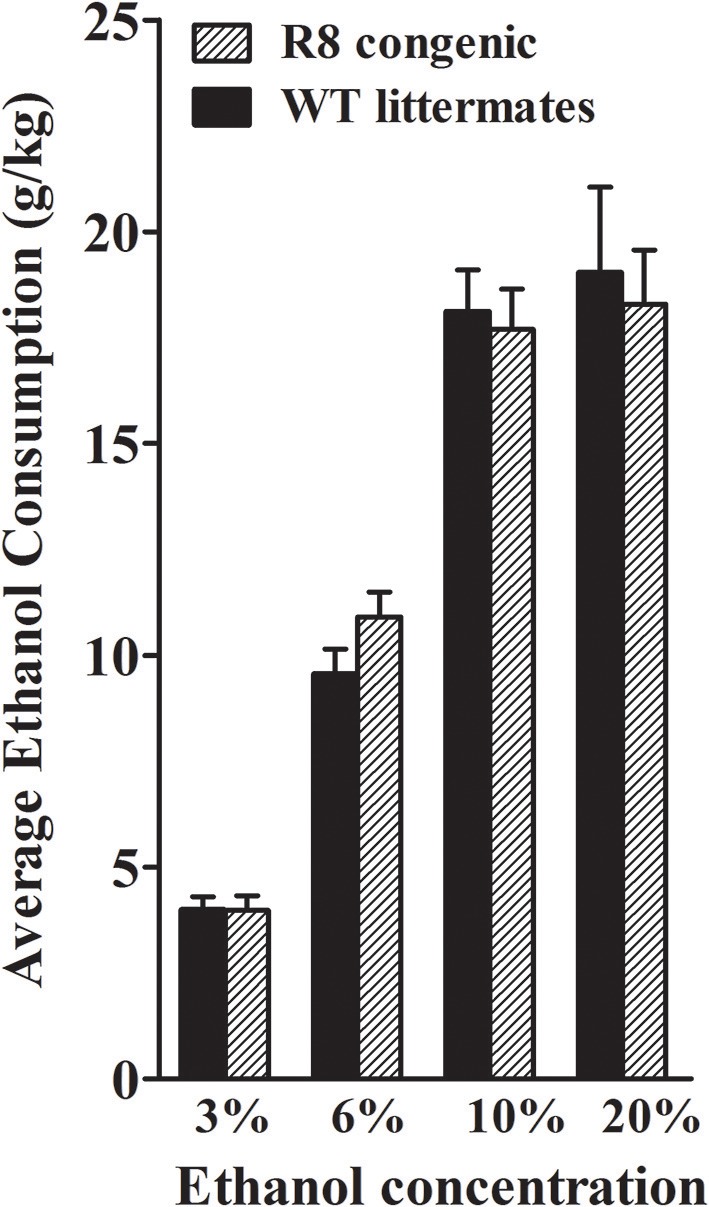
Ethanol consumption does not differ between R8 congenic and WT background strain animals. Ethanol consumption (mean ± SEM) is plotted vs. ethanol concentration offered. There were no differences in the amount of 3, 10, and 20% ethanol consumed between R8 and B6 background strain mice (all *p*'s > 0.7), but there was a trend for R8 mice to drink more 6% ethanol than B6 mice (*p* = 0.13).

Using a two-bottle, free-choice protocol in which mice could choose either water or an ascending series of ethanol concentrations, ethanol consumption was measured in female B6 genetic background congenic (R8) and WT background strain animals. As shown in Figure 9, R8 and WT animals showed comparable consumption of 3, 10, and 20% ethanol solutions [all *t*_(1, 26)_ < 0.3 and *p* > 0.7, NS], with a trend detected for R8 to potentially drink more of the 6% ethanol solution than WT littermates [*t*_(1, 26)_ = 1.6, *p* = 0.13]. R8 and WT animals preferred 3, 6, and 10% ethanol (preference ratios >0.5), but not 20% ethanol (preference ratio < 0.5), compared to tap water (data not shown); with no difference between R8 and WT detected [all *t*_(1, 26)_ < 1.3, *p* > 0.2, NS]. One week after the ethanol drinking study, the same mice were tested for saccharin intake (selected for its sweet taste), quinine (bitter taste), and potassium chloride (salty taste). These substances are non-caloric and are not known for confounding pharmacological effects. Consumption and preference did not differ between R8 and WT animals for any of the tastants (all *p* > 0.1, Supplementary Figure 2), though R8 animals showed a trend to drink more 0.066% saccharin than WT [*t*_(1, 26)_ = 2.0, *p* = 0.054]. There were no differences in water consumption or in total volume of fluid consumed [all *t*_(1, 26)_ < 1.6, *p* > 0.1, NS]. In summary, our results are consistent with the conclusion that *Alcw1*_2_ does not affect alcohol consumption (or preference drinking), and thus does not contribute to the known genetic relationship between this phenotype and alcohol withdrawal in mice (Metten et al., 1998).

### Ethanol drinking and preference (two bottle choice) in *Kcnj9^−/−^* and WT littermates

**Figure 10:**
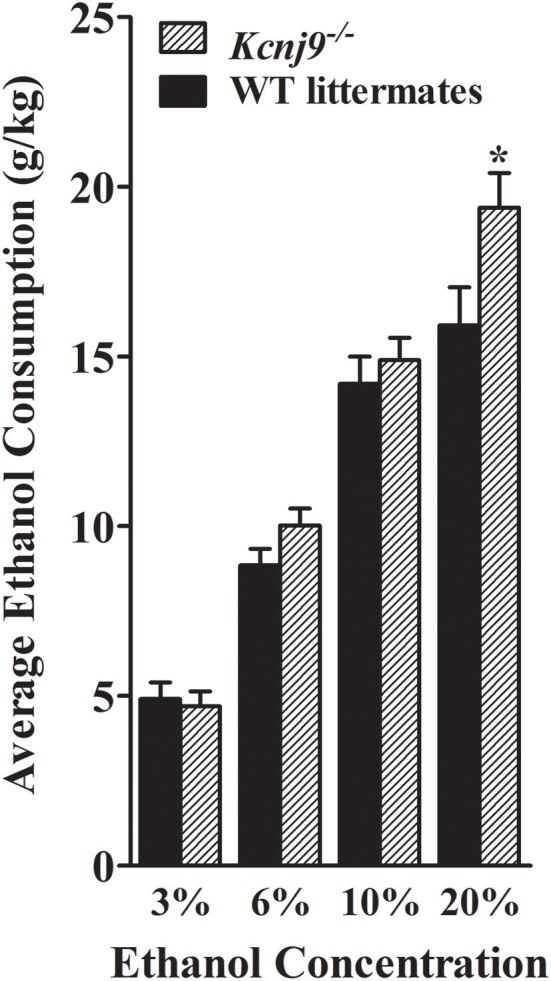
*Kcnj9*^−/−^ mice voluntarily drink more than their *Kcnj9*^+/+^ littermates. Ethanol consumption (mean ±SEM) is plotted vs. ethanol concentration offered. There were no genotype differences between *Kcnj9*^−/−^ and *Kcnj9*^+/+^ mice in 3 or 10% ethanol consumption (*p* = 0.71 and 0.39, respectively) and a trend at 6% (*p* = 0.13). However at 20% ethanol, *Kcnj9*^−/−^ mice drank significantly more ethanol (*p* = 0.014) than WT littermates. *p < 0.05 compared to WT littermates.

Using the same two-bottle, free-choice protocol as described above, ethanol consumption was measured in B6 background *Kcnj9*^−/−^ and WT littermates. A main effect of sex was apparent for each ethanol concentration (3%, *p* = 0.024; 6%, *p* < 2.4 × 10^−11^; 10%, *p* < 2.2 × 10^−11^; 20%, *p* < 2.51 × 10^−11^). However, no GXT interactions were detected (all *p* > 0.3; *n* = 17–22 sex/genotype), therefore the data for both sexes were collapsed to increase statistical power of the analyses. As shown in Figure 10, *Kcnj9*^−/−^ and WT littermates animals showed comparable consumption of 3%, and 10% ethanol solutions [all *t*_(1, 78)_ < 1.4 and *p* > 0.5, NS], with a trend for *Kcnj9*^−/−^animals to drink more 6% ethanol than littermates detected [*t*_(1, 78)_ = 1.4, *p* = 0.16]. *Kcnj9*^−/−^ animals drank significantly more 20% ethanol than WT littermates [*t*_(1, 77)_ = 2.3, *p* = 0.024]. *Kcnj9*^−/−^ and WT littermates preferred 3, 6, and 10% ethanol (all preference ratios >0.5), but not 20% ethanol (preference ratio not different from 0.5), compared to tap water (data not shown); with no difference between *Kcnj9*^−/−^ and WT animals detected for alcohol preference [all *t*_(1, 79)_ < 1.5 and *p* > 0.15, NS]. One week after completion of the ethanol preference drinking studies, the same mice were tested for saccharin, quinine and potassium chloride intake (Supplementary Figure 3). No differences between *Kcnj9*^−/−^ and WT littermates were detected for saccharin consumption or preference at either concentration tested, for quinine consumption at either concentration tested, or for KCl consumption or preference at either concentration tested between genotypes (all *p* > 0.3, NS). Total water consumption and the total volume of fluid consumed also did not differ between *Kcnj9*^−/−^ and WT littermates (both *p* > 0.18, NS).

### Ethanol CPP

**Figure 11:**
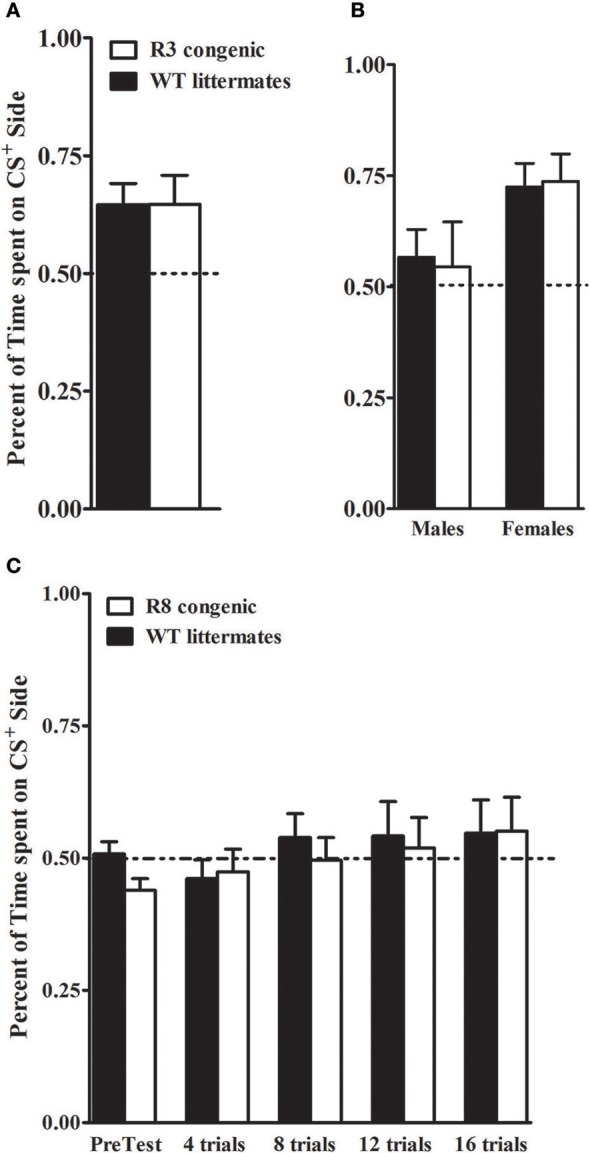
Ethanol CPP in *Alcw1*_2_ congenic and WT littermates. **(A,B)** R3 and WT littermates: on the test day, R3 and WT littermates spent more time on the CS^+^ side than on the CS^−^ side of the testing apparatus with robust ethanol CPP detected in both genotypes (*p* = 0.03 and *p* = 0.005, respectively). Preference is indicated by mice spending > 50% time on the CS^+^ side of the test apparatus. No main effect of genotype (*p* = 0.94) or GXT interaction (*p* = 0.82) were detected. However, a significant difference between males and females is apparent, with females showing robust CPP compared to males (*p* = 0.018; **B**). **(C)** R8 and WT littermates: ethanol CPP was not evident in R8 and WT littermates. The mean time spent on the CS^+^ vs. CS^−^ side of the apparatus did not differ during pretest or during preference tests after 4, 8, 12, or 16 trials (all *p* > 0.27). No main effect of genotype in the time spent on the CS^+^ side (all *p* > 0.23, NS) was detected.

Our recent studies implicate *Kcnj9* as importantly involved in ethanol CPP (Tipps et al., 2016). Therefore, in the present studies, we also assessed this translational phenotype using *Alcw1*_2_ (R3 and R8) congenic and WT animals. Ethanol (2 g/kg, i.p.) induced CPP was robust in R3 congenic and WT littermates [*t*_(1, 30)_ = 4.0, *p* < 5 × 10^−5^, after 8 total conditioning trials, Figure 11A]. This is consistent with work demonstrating significant induction of CPP by 2 g/kg ethanol in D2 strain and D2 genetic background animals (Cunningham et al., 1992; Cunningham, 1995, 2014). Notably, our results show that females exhibited more robust ethanol CPP than males [*F*_(1, 27)_ = 6.3, *p* = 0.018; Figure 11B; *n* = 7–8 sex/genotype]. However, no difference between R3 congenic and WT littermates in ethanol CPP was detected [*F*_(1, 27)_ = 0.0, *p* = 0.94, NS].

B6 genetic background R8 congenic and background strain animals were tested in a separate study. Here, even after 16 conditioning trials and 4 test days, we were unable to detect ethanol CPP (all tests *p* > 0.28, NS, Figure 11C), with comparable results in males and females (all tests *p* > 0.1, NS; *n* = 7–9 sex/genotype). Although a small difference between R8 and WT animals was detected on the pretest day [*F*_(1, 28)_ = 5, *p* = 0.034, with R8 spending less time on the drug-paired floor prior to the conditioning trials], repeated measures ANOVA across the pretest and test days indicated no difference between R8 and WT animals [*F*_(1, 108)_ = 0.34, *p* = 0.55, NS]. Taken together, our results using R3 and R8 *Alcw1*_1_ congenic models are consistent with the conclusion that *Alcw1*_1_ is not involved in ethanol CPP, at least at under the experimental conditions in the present studies.

Table 3 summarizes the alcohol withdrawal and reward phenotypes that have been tested in our genetic models and evidence for a significant role for *Alcw1*_1_ and/or *Alcw1*_1_ is indicated.

**Table 3:** Summary of alcohol phenotypes tested in *Kcnj9/Alcw1*_1_and *Alcw1*_2_ genetic models.

**Alcohol phenotype**	**Genetic model**	**Different vs. WT**	***Evidence for role of***	
			***Alcw1_1_***	***Alcw1_2_***
**Withdrawal (acute or repeated ethanol exposure models)** Enhanced HIC severity	R3 ^a^R8 ^b^R2	++ +++ +++	Yes Yes	Yes
**Withdrawal (chronic model)**			-	
Reduced locomotor activity	R2 R3	(+) +	(Yes)	Yes
Anxiety-like behaviors (EZM)				
Percent time in Open Arms	R2 R3	+ **–**	Yes	No
Number of Open Arm Entries	R2 R3	+ **–**	No	Yes
Number of head dips	R2 R3	(+) +	(Yes)	Yes
**Alcohol-induced CPP**	R3 R8 ^c^*Kcnj9^−/−^*	−− +	No No	Yes
**Alcohol consumption (2 bottle choice)**	R8 *Kcnj9^−/−^*	**–** +	No	Yes

*The alcohol withdrawal and reward phenotypes tested, and the genetic models tested and compared to appropriate WT animals, are indicated. Significant differences between the genetic models tested (R3, R2, or R8 congenic and Kcnj9^−/−^null mutants) and appropriate WT animals are indicated bold and by: +, ++, +++ (for p < 0.05, p < 0.01, and p < 0.001, respectively), with trends (p ~ 0.1) indicated by (+). Evidence for a significant role one or both chromosome 1 QTLs is also indicated as Yes, with trends indicated as (Yes); and evidence indicating no significant role as No. Some of these data are given in detail in recent publications: ^a^(Walter et al., 2017); ^b^(Kozell et al., 2008); ^c^(Tipps et al., 2016). The bolded values indicate that there is a significant difference between genotypes*.

## Discussion

Our data show that two distinct alcohol withdrawal QTLs (*Alcw1*_1_and *Alcw1*_2_) exist on chromosome 1, which account for 13 and 3–6%, respectively, of the genetic variance in acute alcohol withdrawal severity measured using the HIC. Our data also implicate *Alcw1*_1_and *Alcw1*_2_ in withdrawal-induced anxiety-like behavior in alcohol dependent animals, representing the first evidence for their broader roles beyond withdrawal convulsions. Further, we show that this effect is not due to general differences in BEC values. Our data also implicate *Alcw1*_1_ in ethanol consumption and ethanol CPP, but detect no evidence for *Alcw1*_2_ involvement in these reward phenotypes. Our data also point to *Kcnj9* as a high-quality QTG candidate for *Alcw1*_1_. Here, for the first time, we demonstrate using two *Kcnj9*^-/-^ (D2 and B6 background) genetic models, that *Kcnj9*^-/-^ exhibit a robust reduction in alcohol withdrawal severity compared to WT littermates. Additionally, using a B6 background *Kcnj9*^-/-^ genetic model, we demonstrate a modest increase in voluntary alcohol (20%) consumption compared to WT littermates. Thus, our results support a broad role for *Alcw1*_1_*/Kcnj9* in ethanol withdrawal (convulsions and anxiety-like behavior) as well as reward phenotypes. Additionally, our results localize *Alcw1*_2_ to a small 405–923 Kb interval and point to genes involved in mitochondrial respiration as compelling QTG candidates. Thus, our results demonstrate the existence of *Alcw1*_1_and *Alcw1*_2_ as two significant QTLs for alcohol withdrawal convulsions, implicate both in withdrawal-induced anxiety-like behavior, and demonstrate their distinct roles in ethanol-induced CPP and alcohol consumption.

### Withdrawal-induced anxiety-like behavior

Anxiety and anxiety-like behaviors are well-established symptoms of alcohol withdrawal in humans (Driessen et al., 2001) and animal models (Metten et al., 2018), and are thought to affect risk for relapse to alcohol abuse and dependence. Ethanol vapor inhalation has long been used as a tool to induce physical dependence in rodents. In dependent animals, withdrawal-induced anxiety-like behavior can be assessed using a variety of behavioral tests including the open field activity, EZM, elevated plus maze, light-dark box (for review see Kliethermes, 2005) and nesting building (Greenberg et al., 2016). In the present studies animals were tested in the EZM, allowing data collection on multiple measures that interrogate primary and anxiety-like behaviors.

Overall, and particularly for the phenotype percent time in the open arms, our data are consistent with the conclusion that alcohol dependent R2 congenic animals exhibit significantly less severe (and, plausibly, for that reason, shorter duration) withdrawal-induced anxiety-like behavior compared to WT animals. This demonstrates that a gene(s) within the R2 introgressed interval significantly affects withdrawal-induced anxiety-like behavior. Furthermore, the direction of effect is the same direction as for withdrawal convulsions (Walter et al., 2017), supporting the conclusion that the gene(s) affecting withdrawal-induced anxiety-like behavior is, plausibly, the same as that underlying *Alcw1*_1_ or/and *Alcw1*_2_ phenotypic effects on withdrawal convulsions. These data are in agreement and build upon previous work indicating a significant genetic correlation between alcohol withdrawal convulsions and alcohol withdrawal-induced anxiety-like behavior (Metten et al., 2018).

Not surprisingly, given the smaller effect size for withdrawal convulsions apparent in R3 congenic animals, our data using this genetic model are less robust than in the R2 model for withdrawal-induced anxiety-like behavior. Nonetheless, overall, and particularly for phenotypes of numbers of open arm entries and head-dips, our results are consistent with the conclusion that a gene(s) in the smaller R3 introgressed interval is significantly involved in withdrawal-induced anxiety-like behavior. Here again, the direction of effect is the same direction as for withdrawal convulsions, supporting the conclusion that the gene(s) affecting withdrawal-induced anxiety-like behavior is, plausibly, the same as that underlying *Alcw1*_2_ phenotypic effects on withdrawal convulsions. Given that anxiety-like behavioral tests do not necessarily address the same underlying construct (see Milner and Crabbe, 2008), future studies using other anxiety measures to rigorously assess the roles of *Alcw1*_1_ and *Alcw1*_2_in withdrawal-induced anxiety-like behaviors will be important.

### Ethanol CPP

The neural mechanisms that underlie the rewarding effects of ethanol are highly complex. CPP is a widely used measure of drug reward (Sanchis-Segura and Spanagel, 2006). The present studies tested CPP in order to assess the potential role of *Alcw1*_2_in ethanol's rewarding and motivational properties in *Alcw1*_2_congenic and WT animals. Our data show that *Alcw1*_2_congenic and WT animals do not differ in ethanol CPP, and thus do not support a role for *Alcw1*_2_in ethanol CPP. In contrast, our recent data using *Alcw1*_1_ QTG candidate targeted models (*Kcnj9*^−/−^ and WT littermates) show significantly enhanced ethanol CPP compared to WT littermates (Tipps et al., 2016). Further, enhanced ethanol CPP in *Kcnj9*^−/−^ compared to WT littermates is not due to general differences in BECs, the development of ethanol tolerance/sensitization, or the ability of ethanol to alter learning and memory (Tipps et al., 2016). Thus, our data are in agreement and build upon work demonstrating a significant genetic correlation between ethanol CPP and withdrawal (Cunningham, 2014), and also support the involvement of *Alcw1*_1_*/Kcnj9*, but not *Alcw1*_2_, in this genetic relationship.

### Ethanol consumption

The fact that alcohol consumption is a prerequisite for the development of alcoholism is self-evident. In a meta-analysis, Metten et al. (1998) found that low voluntary ethanol consumption using a two bottle choice paradigm is significantly genetically correlated with severe ethanol withdrawal convulsions (using both chronic and acute ethanol exposure models), and *vice versa*, when tested independently in separate animals, suggesting that ethanol consumption and withdrawal may share specific (but anonymous) genetic contributions. Here, we tested the potential role of *Alcw1*_1_and *Alcw1*_2_in ethanol consumption using the two-bottle choice paradigm using female *Alcw1*_2_congenic (R8) and WT animals as well as using male and female *Alcw1*_1_ QTG candidate genetic models (B6 background *Kcnj9*^−/−^ and WT littermates). Our data for ethanol consumption and preference in the WT mice were consistent with levels normally seen in the B6 inbred strain (Belknap et al., 1993; Melo et al., 1996). Overall, our data do not show a role for *Alcw1*_2_ in ethanol consumption using this drinking paradigm. However, our data do indicate that *Kcnj9*^−/−^ mice show a modest increase in ethanol consumption (20%) compared to WT littermates. Our results build upon, but are not entirely consistent with, those of Herman et al. (2015) who reported a significant difference in ethanol consumption using a limited access paradigm, with *Kcnj9*^−/−^ mice consuming more ethanol than WT littermates, but detected no difference using a 15% ethanol two-bottle choice paradigm. However, there are several methodological differences between the two-bottle choice drinking studies. First, Herman et al. (2015) used a single ethanol concentration (15%) and assessed drinking for 6 days, whereas our study used ascending concentrations of ethanol (3, 6, 10, and 20%) for 4 days each. Our study assessed consumption in males and females, whereas Herman et al. (2015) tested only males. We switched sides for presentation of ethanol and water tubes every other day, and use consumption data from days two and four to assess ethanol consumption (Phillips et al., 1994), whereas Herman et al. (2015) switched sides for presentation of ethanol and water daily and did not specify which data was used to assess ethanol consumption. Overall, our data and that of other laboratories indicates a modest but significant difference in ethanol consumption between *Kcnj9*^−/−^ and WT animals.

### Neuronal circuitry activation affected in an *Alcw1* dependent manner

Our data using c-Fos induction as a high-resolution marker of neuronal activation show that mice congenic for a region spanning *Alcw1*_1_ and *Alcw1*_2_ demonstrate significantly (*p* < 0.05) less alcohol withdrawal associated activation than background strain mice in the prelimbic cortex, basolateral amygdala, nucleus accumbens shell, dorsolateral striatum, and caudal substantia nigra pars reticulata (Buck et al., 2017). These data elucidate circuitry by which *Alcw1*_1_ and/or *Alcw1*_2_ influence alcohol withdrawal behaviors. The relative effect sizes for *Alcw1*_1_ and *Alcw1*_2_ suggest a greater influence of *Alcw1*_1_ compared to *Alcw1*_2_ on the brain regions implicated. The prelimbic cortex plays an important role in the inhibition of hypothalamo-pituitary-adrenal (HPA) responses to emotional stress *via* influences on neuroendocrine effector mechanisms (Figueiredo et al., 2003; Radley et al., 2006) and is thought to be involved in ethanol withdrawal behaviors including anxiety-like behavior. Ongoing studies implicate *Kcnj9*/GIRK3 actions in the basolateral amygdala as crucial to alcohol withdrawal-enhanced fear conditioned behavior (Buck and Tipps, unpublished results). Lesions of caudolateral substantia nigra pars reticulata attenuate ethanol withdrawal convulsions and support a role of this brain region in withdrawal convulsions (Chen et al., 2008), with RNA interference (RNAi) analyses demonstrating a role for expression of a different proven alcohol withdrawal QTG (*Mpdz*) on alcohol withdrawal convulsions (Kruse et al., 2014).

### *Alcw1_2_*points to a mechanism involving oxidative homeostasis

Taken together, our analyses using reciprocal R3 and R8 congenic models localize *Alcw1*_2_ to a minimal 405 Kb (maximal 923 Kb) interval on mouse chromosome 1. This region (and the syntenic region in humans) is known for an exceptional gene density, containing 4-5 times more than estimated averages genome wide (Waterston et al., 2002). Moreover, regulation of the expression of genes within this region, as well as mediated by a gene(s) in this region, is complex, involving *cis* and *trans*-regulation (Mozhui et al., 2008; Walter et al., 2017). The present studies finely map *Alcw1*_2_, within which we now delineate eleven genes (*Fcgr3, Tomm40l, Apoa2, Adamts4, B4galt3, Usp21, Nit1, Sdhc, Ndufs2, Ppox*, and *Ufc1*) in the minimal interval that demonstrate *cis*-regulation based on published data (Denmark and Buck, 2008; Walter et al., 2017). Strikingly, three of these genes (*Sdhc, Ndufs2* and *Ppox*) encode proteins involved in mitochondrial oxidative phosphorylation (OXPHOS) pathways (Denmark and Buck, 2008). Furthermore, recent weighted gene coexpression network analyses (WGCNA) using complementary R8 and R2 ISC models implicate an OXPHOS-enriched network module affected by *Alcw1* genotype, and identify *Sdhc* and *Ndufs2* as candidate quantitative trait genes in the OXPHOS co-expression network (Walter et al., 2017). R8 and WT animals differ significantly in ethanol withdrawal severity, but not pentobarbital withdrawal (Kozell et al., 2009), so it is noteworthy that alcohol significantly impacts brain oxidative homeostasis *via* alcohol metabolic by-products which drive OXPHOS and impair the actions of antioxidants (Sun and Sun, 2001; Bailey, 2003), whereas barbiturate exposure has neutral or anti-oxidative actions (Smith et al., 1980; Ueda et al., 2007). Alcohol-induced oxidative damage is well-established, but oxidative status during alcohol withdrawal have been less studied. Nonetheless, rodent studies show increased brain reactive oxygen species for several hours after ethanol exposure (Dahchour et al., 2005) and this correlates well with withdrawal seizure severity (Vallett et al., 1997). Our recent data also demonstrate that N-acetylcysteine, an FDA-approved antioxidant, significantly reduces severity of alcohol withdrawal seizures in mice (Walter et al., 2017). Finally, expression changes for a number of oxidative stress and mitochondrial genes are hallmarks of the human alcoholic brain (Flatscher-Bader et al., 2006; Liu et al., 2006). Thus, although additional genes remain in the *Alcw1*_2_ interval, in our opinion the mounting evidence elevates the status of *Ndufs2, Sdhc*, and *Ppox* as compelling QTG candidate genes.

*Ndufs2* encodes a core Complex I protein (NADH dehydrogenase [ubiquinone] Fe-S protein 2) which is crucial for mitochondrial respiration. *Ndufs2* is significantly DE in reciprocal congenic vs. respective background strains (Walter et al., 2017). Its mRNA content is regulated by ethanol in the amygdala (Most et al., 2015), which is a region implicated in *Alcw1* actions (Buck et al., 2017). In our genetic models, *Ndufs2* also contains a single coding region nonsynonymous SNP that is predicted to be functionally relevant (Denmark and Buck, 2008). Mutation in the *Caenorhabditis elegans* ortholog (*gas-1*) causes oxidative stress (Kayser et al., 2003) and ethanol hypersensitivity (Morgan and Sedensky, 1995), whereas mutations in human *NDUFS2* leads to increased seizure susceptibility (Ugalde et al., 2004). Interestingly, recent data implicates genetic differences in respiratory supercomplex organization, and specifically supercomplexes containing Complex I, in risk for alcohol withdrawal (Buck et al., 2014), suggesting an intriguing mechanism for *Ndufs2* involvement in alcohol withdrawal.

*Ppox* encodes protoporphyrin oxidase (PPOX), which catalyzes the final step of heme biosynthesis, the prosthetic group required for the cytochrome function central to electron transport chain (ETC) activity. Interestingly, there is a mutation in human PPOX (González-Arriaza and Bostwick, 2003) with seizures as a primary symptom. This would be consistent with *Ppox* as a plausible QTG candidate for withdrawal convulsions.

*Sdhc* encodes succinate dehydrogenase complex subunit C (SDHC), a membrane-anchoring subunit that is required for the proper assembly of Complex II in the ETC. *Sdhc* contains multiple functionally critical SNPs between B6/D2 strains (Denmark and Buck, 2008). It shows significant DE between *Alcw1*_2_ congenic and WT animals (Walter et al., 2017), and as noted above for *Ndufs2*, its expression is also regulated by ethanol in the amygdala (Most et al., 2015). *Sdhc* is contained in a significant OXPHOS module in mouse lines selected for the dual traits of alcohol consumption and withdrawal (Metten et al., 2014). In work implicating genetic differences in respiratory supercomplex organization as contributing to differences in alcohol withdrawal risk, Complex II involvement was not apparent (Buck et al., 2014). However, given that Complex II is a convergence point where substrate metabolism is coupled to ATP-generating OXPHOS, *Sdhc* should be considered a high-quality QTG candidate.

### Plausible mechanism involving *Kcnj9*/GIRK3

Our analyses identify *Kcnj9* (GIRK3) as a promising high-quality QTG candidate to underlie *Alcw1*_1_phenotypic effects on alcohol withdrawal symptoms and more. GIRK3 is widely expressed in brain where it contributes to heteromeric GIRK2/3 and GIRK1/3 channels (Torrecilla et al., 2002; Koyrakh et al., 2005; Labouèbe et al., 2007; Ciruela et al., 2010). It is not understood whether GIRK3-containing channels show altered sensitivity to ethanol compared to other GIRK channel subtypes, but GIRK2/3 channels do in fact show reduced sensitivity to Gβγ activation (Jelacic et al., 2000). If GIRK2/3 channels are also less sensitive to activation by ethanol, then the effect of reduced GIRK3 expression could be an enhancement of ethanol's ability to modulate GIRK signaling. Thus, GIRK signaling in *Kcnj9*^−/−^ mice may be more sensitive to modulation by ethanol. Alternatively, reduced GIRK3 expression could affect channel trafficking and thus the adaptation of cells to ethanol exposure. GIRK3 subunits associate with sorting nexin 27 (SNX27), which regulates GIRK channel expression by targeting GIRK3-containing channels to early endosomes. This reduces both cell surface expression of GIRK3-containing channels and GIRK currents (Lunn et al., 2007; Balana et al., 2013). SNX27 itself has also been implicated in the rewarding effects of drugs of abuse (Munoz and Slesinger, 2014), suggesting that the regulation of GIRK signaling *via* this mechanism is an important adaptation to drug exposure. While the effects of ethanol on channel trafficking via SNX27 are unknown, it is possible that this mechanism could play a role in adapting to ethanol exposure and might contribute to the altered dopamine signaling observed following repeated ethanol exposure (Perra et al., 2011; Herman et al., 2015). Future investigations into these possibilities may help address the question of how reduced *Kcnj9* expression and the loss of GIRK3 can alter ethanol responses. Nevertheless, our data and findings from other laboratories support the hypothesis that GIRK channels play an important role in ethanol actions, and suggest that GIRK-based therapeutics, particularly those targeted to specific GIRK subunits, could be effective treatments for alcohol addiction and relapse (Sugaya et al., 2012; Bodhinathan and Slesinger, 2014; Herman et al., 2015; Munoz et al., 2016; Glaaser and Slesinger, 2017).

### Human relevance of QTLs/QTGs identified in mice

Our studies precisely localize *Alcw1*_1_ and *Alcw1*_2_ to a region syntenic with human 1q23.1-23.3. Several studies have identified markers on human 1q associated with alcoholism (reviewed by Ehlers et al., 2010) that, while localized to large regions, are potentially syntenic. However, homology to human remains to be proven. It is worth noting that human studies have generally sought markers associated with the diagnosis and endophenotypes (maximum drinks, metabolism, brain oscillations) rather than withdrawal risk or other phenotypes studied in our animal studies. Two human studies have identified alcohol dependence QTLs (LODs > 3) on the q-arm of human chromosome 1 (Dick et al., 2002; Hill et al., 2004). A third human QTL for tobacco usage has been identified in this same region (Ehlers and Wilhelmsen, 2006) and a fourth on 1q for heavy drinking (Guerrini et al., 2005). Finally, there is evidence from a family-based association study (Hill et al., 2013) for an alcohol dependence QTL on 1q. A recent genome-wide association study (GWAS) by Zuo et al. (2012) also detected variants associated with alcohol dependence on 1q, and depending on what correction method they used, alcohol dependence was associated with variants in or very near the *Alcw1*_1_*/Alcw1*_2_ syntenic interval, just missing the cutoff threshold. This group identified KIAA0040 as a plausible candidate. However, the majority of the subjects were co-dependent on nicotine, and nearly half were co-dependent on cocaine and marijuana. Further, the same group finds that this same locus is significantly associated with nicotine-alcohol co-dependence (Zuo et al., 2012), suggesting that its influence may not be specific (or even related) to alcohol dependence. Furthermore, the authors state that nearly half of the subjects were co-dependent on cocaine and marijuana. Interestingly, a recent publication (Han et al., 2013) that examined the protein interaction networks associated with alcohol dependence [using the same SAGE and COGA datasets used by Zuo et al. (2012)] also finds hits on 1q are just shy of the cutoff threshold used, and may be syntenic to *Alcw1*_1_/*Alcw1*_2_. Thus, while it is true that the relevance of *Alcw1*_1_ and *Alcw1*_2_ to alcohol dependence in humans is not certain, the currently available data do not imply evidence against it either.

### Summary

We have now confirmed five significant ethanol withdrawal QTLs, i.e., two on chromosome 1 (*Alcw1*_1_ and *Alcw1*_2_), one on chromosome 4 (*Alcw2*, for which we have identified *Mpdz* as a causal QTG; Milner et al., 2015; Kruse et al., 2014), one on chromosome 11 (*Alcw3*; Buck et al., 1997; Hood et al., 2006), and one on chromosome 19 (Buck et al., 2002). At least three of these (*Alcw1*_1_, *Alcw1*_2_, and *Alcw2*/*Mpdz*) are now implicated as having distinct broader roles in alcohol actions, including reward phenotypes [Milner et al., 2015, and unpublished results]. Interestingly, our data may also point to synergistic mechanisms involving oxidative homeostasis and GABA receptor function. Ongoing work using *Mpdz* genetic models points to its actions affecting GABA_B_ receptor function (Kruse and Buck, unpublished results) and OXPHOS (Walter and Buck, unpublished results) and thus might act synergistically with *Alcw1*_1_*/Kcnj9* and/or *Alcw1*_2_. The possibility that the genes underlying *Alcw1*_1_ and *Alcw1*_2_ may play an important role in distinct translational responses makes them important targets.

## Author contributions

DD, NW, LK, and KB participated in writing and revising the manuscript. NW, LK, and KB participated in the concept and design of these studies. LK acquired and analyzed the data. DD, NW, and LK were involved in interpretation of the data.

### Conflict of interest statement

The authors declare that the research was conducted in the absence of any commercial or financial relationships that could be construed as a potential conflict of interest. The contents of this publication do not represent the views of the U.S. Department of Veterans Affairs or the United States Government.
