# Supplementary material for: Distinct Roles for Two Chromosome 1 Loci in Ethanol Withdrawal, Consumption, and Conditioned Place Preference
Source: Front Genet. 2018 Aug 27;9:323. doi: 10.3389/fgene.2018.00323 (PMC6120100; doi:10.3389/fgene.2018.00323)
Supplement: Supplementary file 2 [file Presentation_1.pdf]

Supplementary Material

Distinct roles for two chromosome 1 loci in ethanol withdrawal, consumption, and conditioned place preference

Laura B. Kozell*, Deaunne L. Denmark, Nicole A.R. Walter, Kari J. Buck

*** Correspondence:** Kari J. Buck: buckk@ohsu.edu

Supplementary Tables.

Supplementary Table 1

Blood Ethanol Concentrations (mg/ml) during 3 day vapor-chamber exposure

Experimental Test	Strain or Treatment	Day	N	Average BEC (mg/kg)
**Elevated Zero Maze**	**R2 congenic**	1	16	1.15±0.06
		2	16	1.40±0.05
		3	16	1.48±0.06
	**WT background strain**	1	14	1.05±0.08
		2	14	1.24±0.07
		3	14	1.41±0.10
**Elevated Zero Maze**	**R3 congenic**	1	17	1.29±0.04
		2	16	1.53±0.07
		3	16	1.31±0.09
	**WT littermates**	1	16	1.34±0.04
		2	16	1.44±0.06
		3	16	1.21±0.06

Sample size is indicated by *n*.

Table 2. R2 congenic and WT background strain behavioral measurements in EZM

Time Point	Time in Open Arms	Open Arm Entries	Head Dips	Activity (cm/10 min)
**WT background Ethanol group (n=14)**				
**Habituation Day**				
1	201±19	70±6.1	15.8±1.8	4093±147
2	131±10	47±4.5	10.1±1.4	3908±159
3	90±7.7	38±6.3	12.4±2.2	3167±172
**Post Ethanol**				
7h	37±12	13±4.6	1.7±0.9	1844±165
24h	114±15	29±4.5	9.3±1.6	2605±268
48h	96±13	32±5.7	8.4±1.7	2536±171
**WT background Air-group (n=11)**				
**Habituation Day**				
1	195±22	72±12	19.0±4.2	4013±250
2	125±13	57±6.9	12.1±1.2	3728±282
3	72±6	44±6.7	7.3±1.3	3086±153
**Post Ethanol**				
7h	166±20	55±6.1	19±2.0	3655±80
24h	177±25	60±9.9	19±3.1	3630±306
48h	145±21	35±3.8	19±3.1	2885±151
**R2 congenic Ethanol group (n=15)**				
**Habituation Day**				
1	164±22	64±8.6	12.4±1.8	3637±127
2	140±14	48±5.7	14.1±1.6	3358±102
3	118±13	39±4.9	15.3±2.0	2722±103
**Post Ethanol**				
7h	71±13	22±5.0	3±0.6	1953±68
24h	138±13	38±3.5	10.8±1.8	2862±226
48h	135±12	44±4.5	13.4±1.8	2825±145
**R2 congenic Air-group (N=7)**				
**Habituation Day**				
1	172±28	56±7.2	13.1±1.7	3959±150
2	153±18	54±7.2	14.2±1.7	3346±203
3	100±16	33±5.5	12.4±2.5	2712±194
**Post Ethanol**				
7h	131±12	60±12	15±2.2	3528±164
24h	123±13	48±10	17±1.9	2944±258
48h	127±13	35±4.5	19±4.3	2669±116

Values shown represent the mean ± SEM.

Supplementary Table 3. R3 congenic and littermate mice behavioral measurements in EZM

Day	Time in Open Arms (sec/10min)	Open Arm Entries	Head Dips	Activity (cm/10min)
**WT Littermates Ethanol group (n=15)**				
**Habituation Day**				
1	180±17	48±3.8	14.4±2.5	2779±99
2	150±12	37±2.8	12.4±2.3	2573±147
3	132±10	34±2.7	11±1.7	2557±124
**Post Ethanol**				
24h	132±22	22±2.6	4.9±1.4	2032±121
48h	144±22	31±4.2	8.9±2.2	2177±83
**WT Littermates Air-group (n=9)**				
**Habituation Day**				
1	192±13	48±3.9	14.8±1.9	2837±66
2	164±17	40±5.4	11.8±1.9	2498±145
3	138±9	36±3.3	11.7±1.1	2684±123
**Post Ethanol**				
24h	146±19	40±6.3	15.9±2.7	2740±145
48h	143±19	46±5.6	16.9±2.3	2642±160
**R3 Congenic Ethanol group (n=20)**				
**Habituation Day**				
1	209±14	50±3.0	15.7±1.2	2797.±80
2	149±13	28±2.4	12.4±1.6	2621±125
3	127±9	32±2.8	11.0±1.5	2557±99
**Post Ethanol**				
24h	155±22	26±2.5	7.4±1.7	1943±131
48h	152±18	34±3.3	11.1±2.0	2343±109
**R3 Congenic Air group (n=13)**				
**Habituation Day**				
1	203±15	56±5.6	14.2±1.7	2987±233
2	125±12	27±2.3	9.2±2.0	2586±159
3	108±16	25±2.5	8.5±2.6	2347±160
**Post Ethanol**				
24h	139±17	31±3.1	9.3±1.6	2559±165
48h	118±14	29±3.9	8.8±1.7	2381±93

Values shown represent the mean ± SEM.

1.1 Supplementary Figures

Supplementary Figure 1.

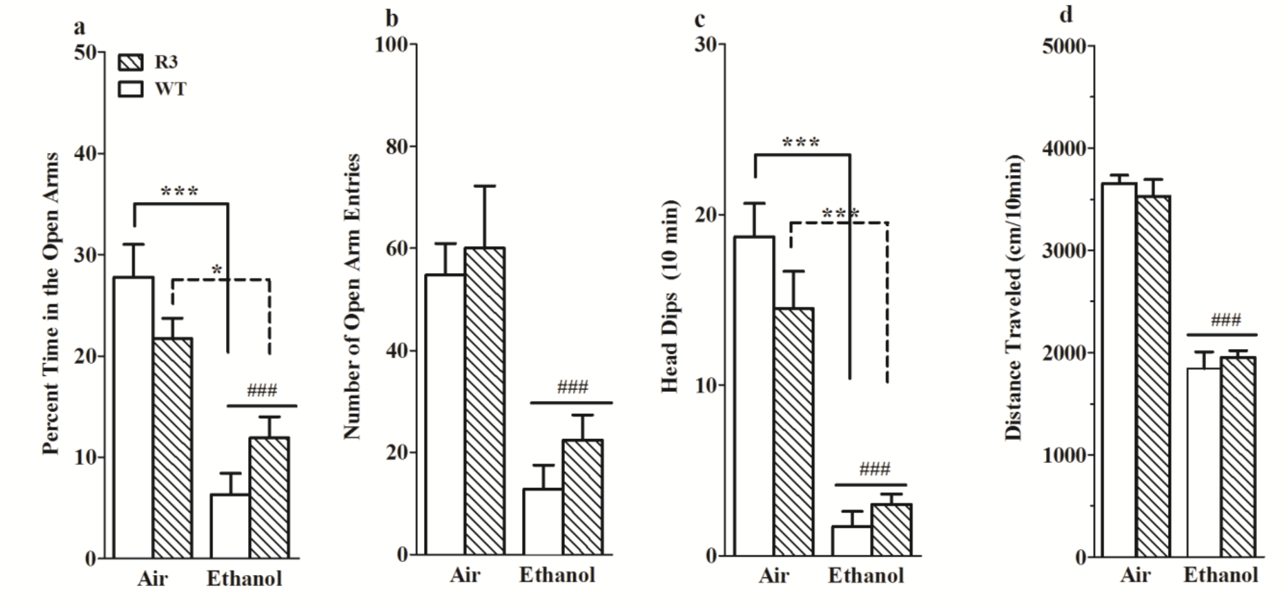

Supplementary Figure 1 (S1). Anxiety-like behavior during withdrawal in *$Alcw1_1$* congenic R2 and WT animals. Panels show the (a) total distance traveled (mean ± SEM), (b) % open time, (c) number of open entries and (d) head dips on the EZM during a 10 min test. A main effect of treatment was evident 7 h post ethanol with ethanol withdrawn animals exhibited **(a)** a reduction in the distance travelled ($F_{1,43} = 168$, $p < 2\text{x}10^{-11}$), and robust (63%) reductions in the **(b)** percent time spent in the open arms ($F_{1,42} = 37.4$, $p < 4\text{x}10^{-7}$), **(c)** open arm entries ($F_{1,42} = 37.1$, $p < 3\text{x}10^{-7}$) and **(d)** head dips compared to air-controls ($F_{1,42} = 118$, $p < 2.1\text{x}10^{-11}$). There were no main effects of genotype (all p > 0.3). There were no significant GXT interactions in **(a)** distance travelled ($F_{1,43} = 0.8$, $p = 0.37$) or **(c)** open arm entries ($F_{1,42} = 0.11$, $p > 0.7$, **Figure S1c**), however there were significant GXT interactions in the **(b)** percent time spent in the open arms ($F_{1,39}$=5.0, $p = 0.032$), and **(d)** head dips ($F_{1,42} = 4.4$, $p = 0.042$), with both ethanol withdrawn WT and R2 spending less time in the open arms ($p = 7\text{x}10^{-6}$ and $p = 0.049$, respectively) and making fewer head dips than their respective air control animals (both $p < 8\text{x}10^{-6}$).

Supplementary Figure 2.

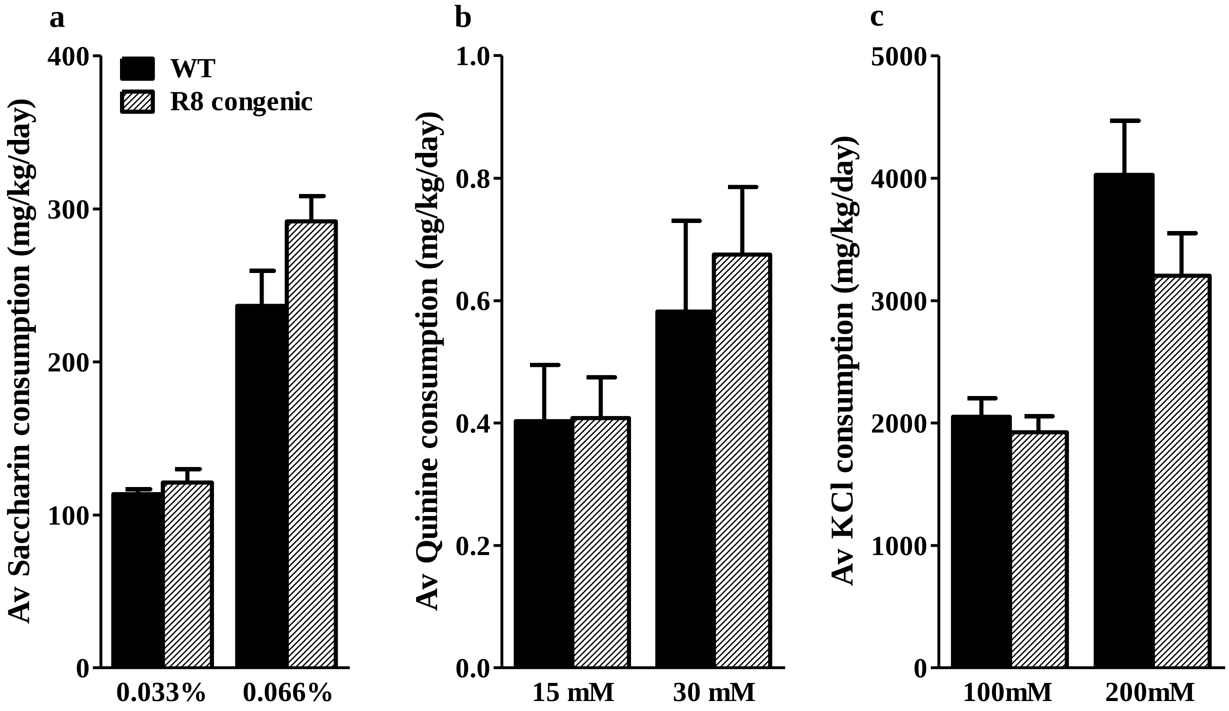

Supplementary Figure 2 (S2). Saccharin, Quinine and KCl consumption in R8 congenic and WT background strain mice. Consumption and preference did not differ between R8 and WT animals for saccharin, quinine or KCl (all $p > 0.1$) except for the 0.066% saccharin solution ($t_{1,26} = 2.0$, p = 0.054). R8 congenics showed a trend to drink more 0.066% saccharin than WT littermates.

Supplementary Figure 3.

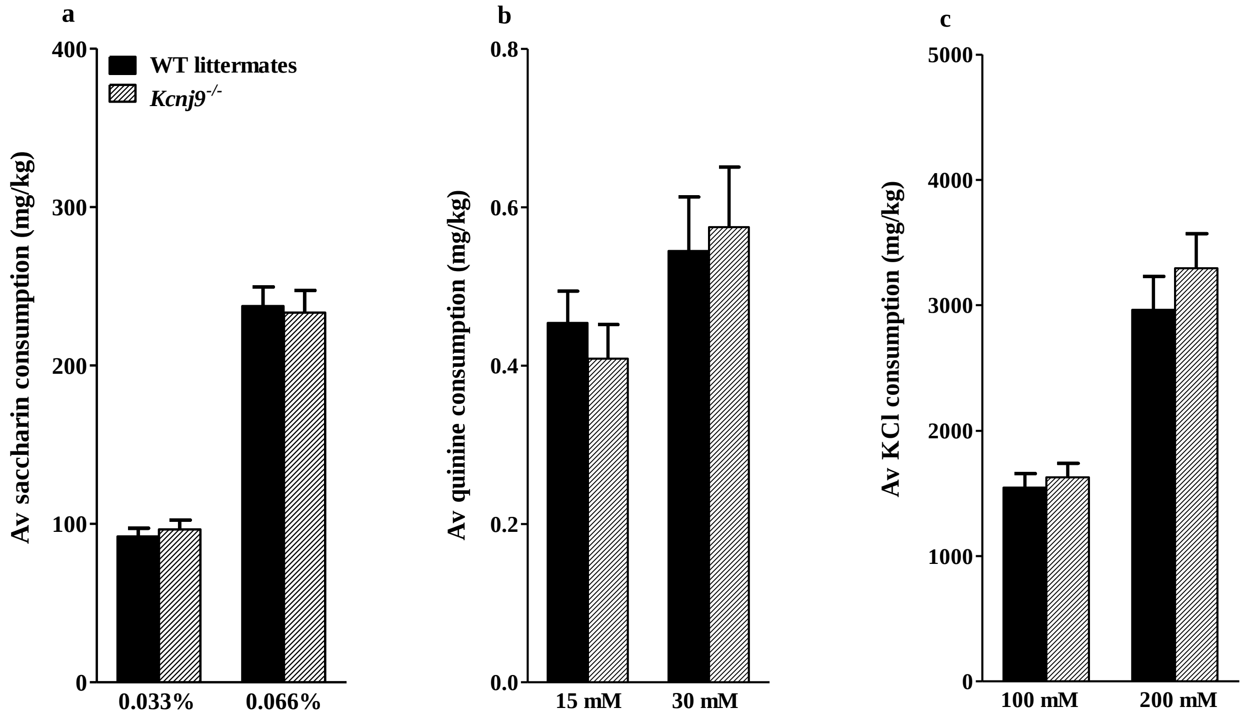

Supplementary Figure 3. Saccharin, quinine and KCl consumption in *Kcnj9*$^{-/-}$ and WT littermate mice. Consumption and preference did not differ between *Kcnj9*$^{-/-}$ and WT littermates for saccharin, quinine and KCl consumption (all $p \geq 0.3$, NS). Total water consumption and the total volume of fluid consumed also did not differ between *Kcnj9*$^{-/-}$ and *Kcnj9*$^{+/+}$ littermates (both $p >$ 0.18, Data not shown).
